# Shared and distinct cognitive processes across multiple virtual navigation tasks

**DOI:** 10.1016/j.isci.2026.117592

**Published:** 2026-09-24

**Authors:** Varnan Chandreswaran, David Stawarczyk, Anne Bierbrauer, David Clewett, Iva Brunec, Gillian Coughlan, Lila Davachi, Hugo Spiers, Michael Hornberger, Marta Silva, Lluis Fuentemilla, Morgan Barense, Jason Ozubko, Lukas Kunz, Dorothea Pink, Nikolai Axmacher, Hui Zhang

**Affiliations:** 1 Department of Neuropsychology, Institute of Cognitive Neuroscience, Faculty of Psychology, Ruhr University Bochum, 44801 Bochum, Germany; 2 Psychology and Neuroscience of Cognition Research Unit, University of Liège, Place des Orateurs 1 (B33), 4000 Liège, Belgium; 3 GIGA – CRC (Cyclotron Research Center) Human Imaging, University of Liège, Allée du 6 Août 8 (B30), 4000 Liège, Belgium; 4 Institute of Systems Neuroscience, Medical Center Hamburg-Eppendorf, Hamburg, Germany; 5 Department of Psychology, University of California, Los Angeles, Los Angeles, CA 90095, USA; 6 Department of Psychology, University of Pennsylvania, Philadelphia, PA 19104, USA; 7 Department of Neurology, Massachusetts General Hospital, Harvard Medical School, Boston, MA, USA; 8 Department of Psychology, Columbia University, New York, NY 10027, USA; 9 Institute of Behavioural Neuroscience, Division of Psychology and Language Sciences, Department of Experimental Psychology, University College London, London WC1H 0AP, UK; 10 Norwich Medical School, University of East Anglia, Norwich NR4 7TJ, UK; 11 Cognition and Brain Plasticity Group, Bellvitge Institute for Biomedical Research, 08907 L’Hospitalet de Llobregat, Spain; 12 Department of Cognition, Development and Educational Psychology, University of Barcelona, 08035 Barcelona, Spain; 13 Institute of Neurosciences, University of Barcelona, 08035 Barcelona, Spain; 14 Department of Psychology, University of Toronto, Toronto, Ontario M5S 3G3, Canada; 15 Rotman Research Institute at Baycrest, Toronto, Ontario M6A 2E1, Canada; 16 Department of Psychology, State University of New York at Geneseo, Geneseo, NY 14454, USA; 17 Department of Epileptology, University Hospital Bonn, Bonn, Germany

**Keywords:** spatial navigation, virtual navigation, path integration, spatial memory, wayfinding, event boundaries, individual differences

## Abstract

Although virtual navigation tasks are widely used to investigate human spatial navigation, the processes underlying behavior across tasks remain poorly understood. To address this gap, we administered four virtual navigation tasks assessing spatial learning, path integration, and wayfinding in a single cohort. Participants additionally completed four event segmentation tasks to examine links with episodic memory. Across tasks, spatial cues such as landmarks or the arena center improved performance, with better outcomes when navigating near these cues and when goals were nearby. Despite these shared effects, between-task correlations were sparse. Significant associations emerged between the two path integration tasks, whereas reliance on spatial cues showed little correspondence. However, principal component analysis identified two behavioral dimensions: one aligned with self-reported preference for scene-based navigation, the other with reduced route-based navigation. Episodic memory showed no association with navigation. Virtual navigation tasks thus capture partially distinct processes, and dimensionality reduction helps disentangle navigation mechanisms.

## Highlights

• Spatial cues improved performance across virtual navigation tasks
• Reliance on spatial cues showed little correspondence across tasks
• Path integration showed the strongest cross-task overlap
• PCA revealed two dimensions of virtual navigation behavior

## Graphical abstract

**Figure:**
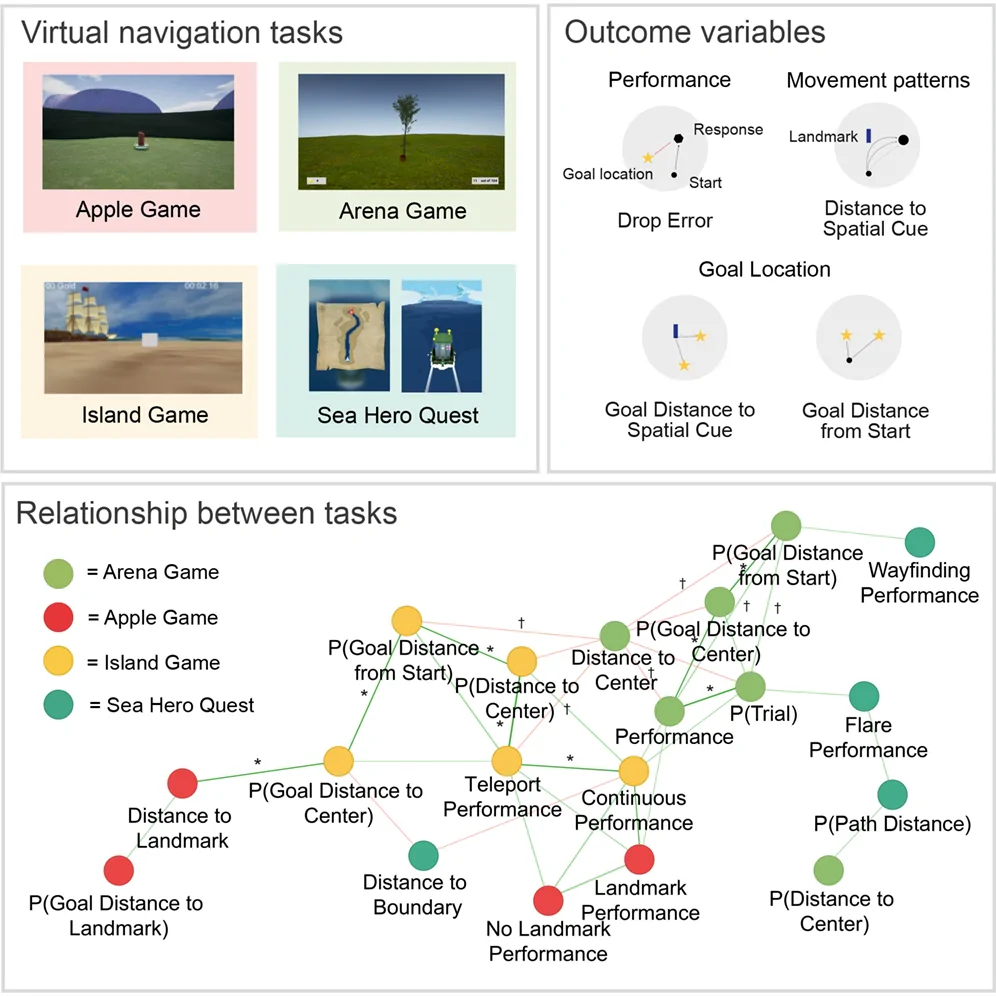

## Teaser

Natural sciences; Biological sciences; Neuroscience; Cognitive neuroscience

## Introduction

Spatial navigation is not a unitary cognitive function but is assumed to rely on multiple processes, including path integration and strategies such as route-based and map-based navigation. Path integration refers to the continuous integration of self-motion cues to maintain an estimate of one’s position and orientation.^1^^,^^2^ At the same time, individuals may learn the environment and strategically rely on spatial cues, such as landmarks, either to guide route-based navigation through sequences of actions or to construct a cognitive map of the environment.^3^ Animal research suggests that hippocampal-entorhinal circuits, including place cells, grid cells, head direction cells, and boundary cells, integrate both idiothetic signals (e.g., vestibular and proprioceptive input) and allothetic signals (i.e., external sensory input) to support estimates of spatial position and orientation.^4^^,^^5^^,^^6^

In recent years, video games have become a popular tool for studying human behavior.^7^ In the domain of spatial navigation, these approaches involve participants navigating simulated environments (Figure 1). One example is the wayfinding task “Sea Hero Quest”, which requires participants to use a map to navigate a sequence of checkpoints.^8^ Another task is the “Arena Game”, a spatial learning task in which participants have to remember the locations of objects in a virtual environment.^9^^,^^10^^,^^11^ The “Apple Game” is a homing task in which participants navigate a path to find an apple and then return to the starting point.^12^^,^^13^^,^^14^ In some trials, the presence of a landmark or boundary provides spatial cues and reliably improves performance. Finally, a task called the “Island Game” also assesses homing, but introduces a teleportation manipulation in which participants are relocated to a different position, requiring them to reorient before continuing the task. Because these virtual navigation tasks are performed on a desktop or mobile device, they largely lack idiothetic self-motion signals and instead rely predominantly on allothetic visual information, such as optic flow and environmental cues, to guide navigation.

**Figure 1:**
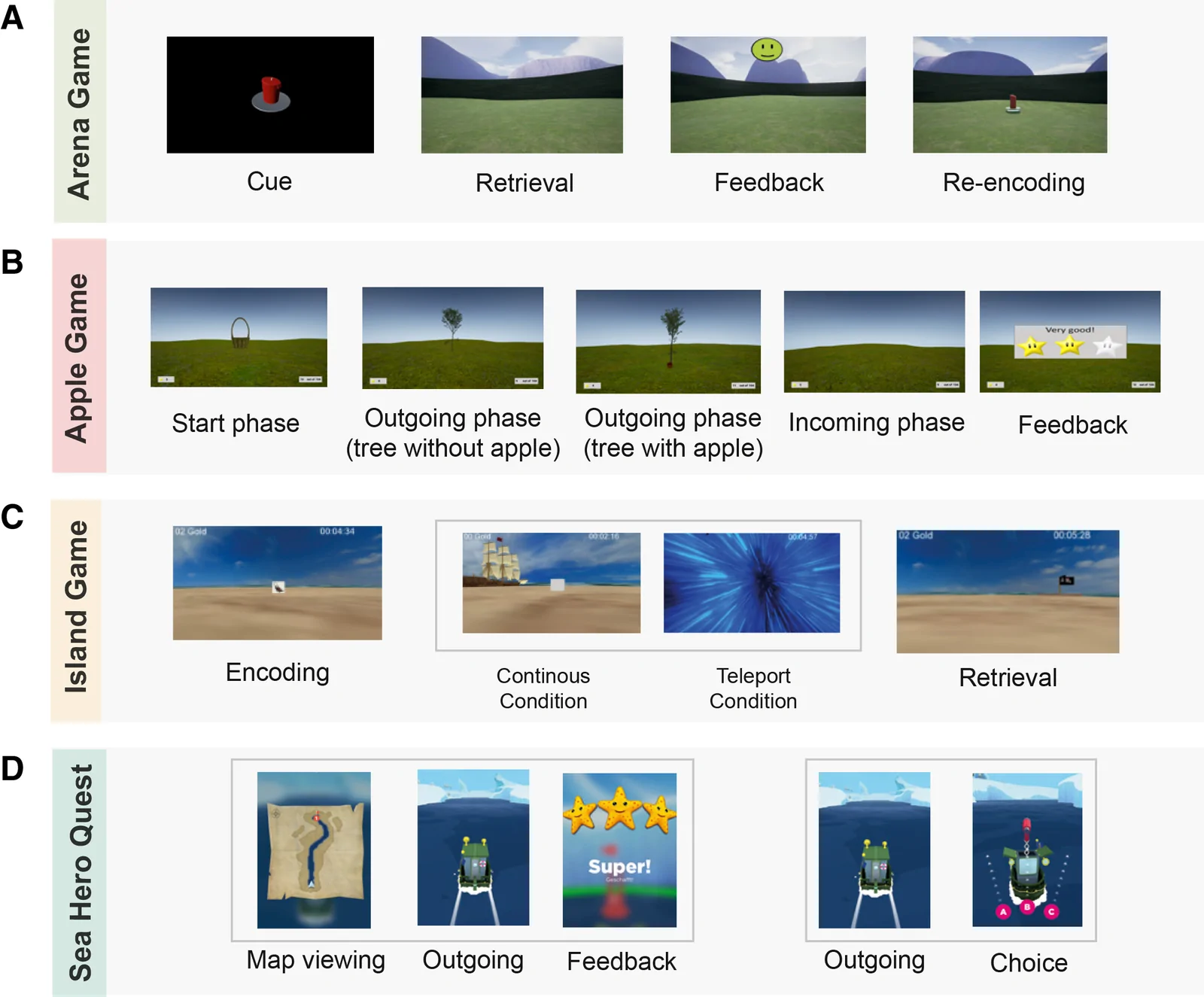
Experimental design of the four spatial navigation tasks (A) Arena Game: participants performed an object-location memory task while navigating freely in a circular virtual arena. At the beginning of each trial, an everyday object was presented (“cue”) which had to be placed as accurately as possible at its correct location during retrieval (“retrieval”). Participants received feedback via smiley faces (“feedback”) and re-encoded the object position afterward (“re-encoding”). (B) Apple Game: in each trial, participants collected a basket (“start phase”) and tried to remember its location (goal location). After navigating toward a variable number of trees (1–5; “outgoing phase”), which disappeared after having been reached, participants had to find their way back to the goal location (“incoming phase”). Lastly, they received feedback via different numbers of stars, depending on response accuracy. (C) Island Game: during the encoding phase, an image appeared on the island and participants collected it by navigating to its location. Participants were encouraged to remember details about the image and its location. Subsequently, their spatial memory was assessed. They were either teleported to a new location or directed to navigate continuously to a new location indicated by an empty cube. From the new location, they navigated to the goal location. (D) Sea Hero Quest: participants performed gamified wayfinding and path integration tasks. The wayfinding component required memorizing a map (“map viewing”) and navigating to checkpoints (“outgoing/feedback”). The path integration component involved navigating a trajectory and subsequently indicating the direction of the starting point (“choice”).

In addition to virtual navigation tasks, researchers also use questionnaires to assess self-reported navigational strategies. One questionnaire measures the extent to which people use map-based strategies rather than scene-based strategies when navigating in the real world (NSQ14^15^). Another questionnaire assesses map-based, route/procedural strategies and egocentric updating (NSQ44^16^).

Despite their widespread use, there is limited consensus on the specific cognitive processes these tasks measure and how they relate to one another.^17^^,^^18^^,^^19^ Since most tasks do not probe specific processes, it is often unclear which processes contribute to navigation behavior.^20^ Even tasks that seemingly rely on similar processes can differ substantially. This raises questions about the convergent validity of navigation measures, which is the degree to which different tests that claim to measure the same construct show agreement. A further complication is that navigation performance is influenced not only by cognitive processes but also by contextual and state-dependent factors, including stress, familiarity with the task, gaming experience, upbringing, and sleep.^14^^,^^21^^,^^22^^,^^23^ It therefore remains unclear to what extent performance in virtual navigation tasks reflects underlying navigation processes as opposed to task-specific or situational influences.

Recent work has begun to apply dimensionality reduction approaches to distinguish common navigation dimensions from variance introduced by specific tasks. Malanchini et al.^24^ applied factor-analytic methods to a large battery of spatial tasks and identified three substantially correlated factors, navigation, object manipulation, and visualization, which in turn loaded strongly onto a higher-order general spatial ability factor. Using confirmatory factor analysis across multiple navigation tasks, including one real-world and two virtual environments, Lader et al.^17^ examined performance in map-building, landmark pointing, and route-efficiency tasks and showed that navigation performance reflects both shared variance across tasks and substantial task-specific variation. Similarly, Topete et al.^25^ analyzed virtual navigation tasks involving guided route learning followed by map reconstruction, landmark pointing, and efficient wayfinding, and found that performance is best captured by two highly correlated factors distinguishing learning in large, open environments from smaller, grid-like environments. Together, these findings highlight the value of dimensionality reduction approaches for capturing both shared and task-specific components of navigation ability and for characterizing nuanced individual differences in navigation behavior.

Beyond characterizing structure within navigation, dimensionality reduction approaches may also help address whether navigation shares common processes with other cognitive domains. Emerging work links spatial navigation and episodic memory, with both relying on medial temporal lobe structures.^26^^,^^27^ Some suggest that the hippocampal-entorhinal system encodes a cognitive map in which grid-like codes represent relational structures, supporting flexible application to both spatial and non-spatial domains.^28^^,^^29^ There is also behavioral evidence consistent with this link. For example, Fragueiro et al.^30^ found that training in egocentric navigation based on path integration selectively improved episodic memory specifically order memory performance, but not semantic or short-term memory, suggesting a possible causal relationship between egocentric navigation mechanisms and episodic memory. Importantly, Nguyen et al.^27^ applied dimensionality reduction to both spatial and episodic memory measures in a naturalistic paradigm and found that they loaded on two highly correlated latent factors, suggesting partially shared higher-order structure across domains.

Another convergence is the role of event boundaries, which are contextual shifts that segment continuous experience into discrete events and shape episodic memory.^31^^,^^32^ In a sequential learning paradigm, event boundaries have been induced by switching auditory contextual cues from one ear to the other while participants encoded a sequence of items, disrupting temporal order memory, increasing perceived temporal distance between items, and enhancing item-context memory. Converging evidence suggests that similar boundary signals arise during navigation.^33^ In the Bus Game, a virtual route-learning task in which participants navigate a city environment, encode intersections along a predefined route, and subsequently perform recognition judgments, turns along the route serve as event boundaries and enhance memory for route elements.^34^^,^^35^^,^^36^ Because of the similarities, one account argues there might be a shared boundary detection mechanism supporting both navigation and episodic memory.^33^

The aim of this study was to determine which aspects of behavior generalize across virtual navigation tasks and which are task-specific by testing a single cohort across multiple tasks and relating these measures to the episodic domain (see Figure 2). Specifically, we examined behavior in four virtual navigation tasks: the Arena Game, Apple Game, Island Game, and Sea Hero Quest.^8^^,^^9^^,^^12^^,^^37^ Across these tasks, we quantified not only overall navigation performance but also several behavioral indices capturing reliance on spatial cues. These included the effects of cue availability (e.g., landmarks or boundaries), movement relative to landmarks, boundaries, or the arena center, and goal distance to spatial cues or the start location. To test whether these measures reflect a common underlying process, we applied dimensionality reduction and examined associations with self-reported navigation strategies.^15^^,^^16^

**Figure 2:**
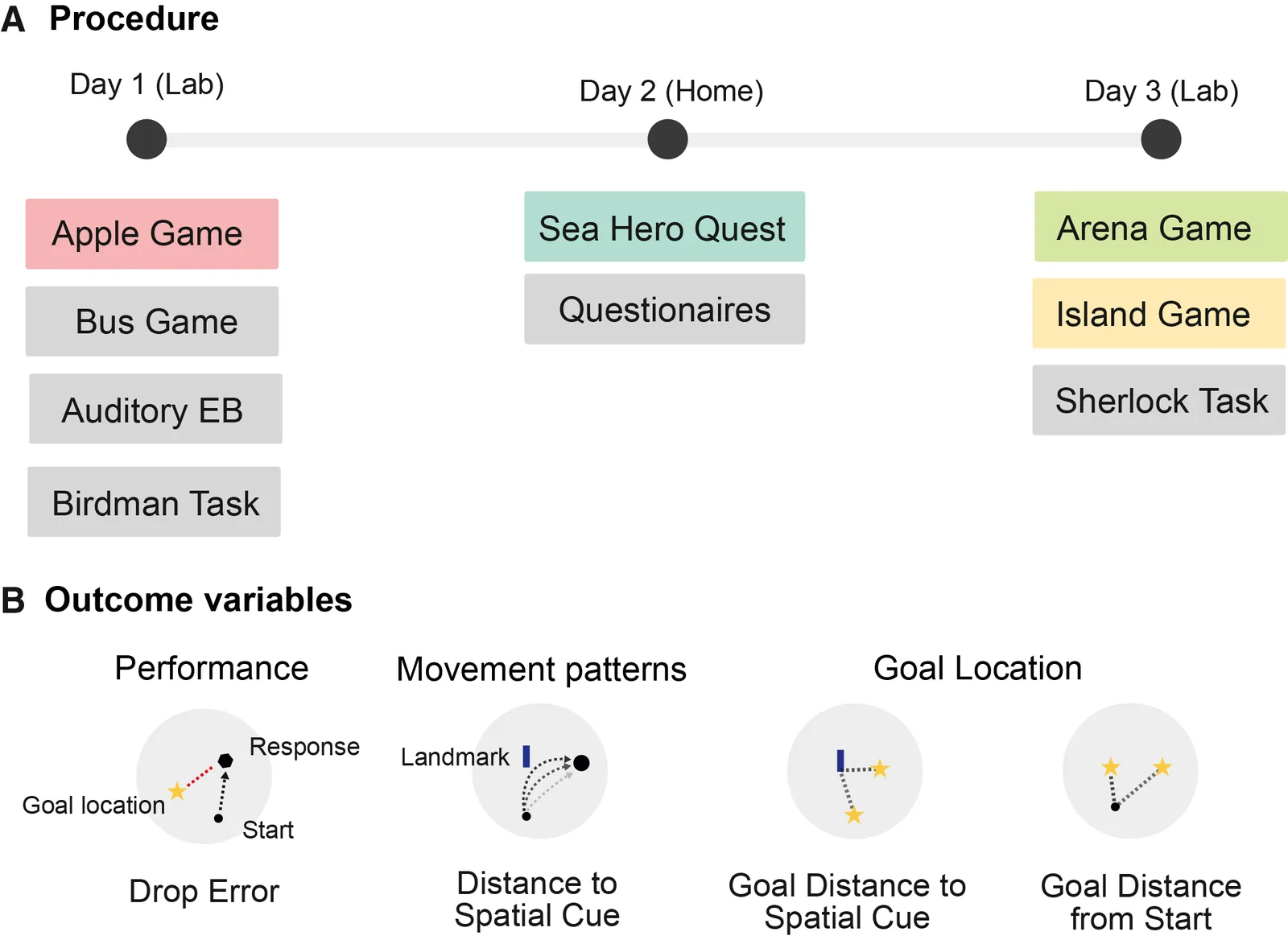
Experimental procedure and calculation of navigation variables (A) Overview of the three-day study protocol. On day 1, participants completed the Apple game, Bus Game, auditory event boundary (auditory EB) task, and Birdman task in the laboratory. Day 2 consisted of home-based testing, including selected levels of Sea Hero Quest and completion of questionnaires. On day 3, participants returned to the laboratory to complete the Arena Game, Island Game, and Sherlock task. (B) Schematic illustration of the primary navigation metrics. Performance was quantified as drop error, defined as the Euclidean distance between the true goal location (yellow star) and the participant’s response location (black dot). Movement patterns were characterized by the average distance of the participant’s trajectory (dotted line) to spatial cues (e.g., landmarks). Goal-location metrics included the distance of the goal relative to spatial cues and the distance of the goal from the starting position.

Participants additionally completed a set of episodic memory tasks targeting the encoding and retrieval of temporal structure, contextual information, and recognition, as well as the effects of event boundaries on these memory outcomes. They completed a sequential learning task in which they encoded sequences of object images and were later tested on order memory, temporal distance estimation, item-context memory, and the effects of auditory event boundaries. They also completed two movie-based tasks that assessed order memory, temporal distance, and recognition, as well as how event boundaries, in this case changes between scenes, influenced these measures. Finally, participants completed the Bus Game, in which recognition memory was assessed for scenes of bus stops encountered along a route, with turns at intersections after bus stops serving as event boundaries.^32^^,^^34^^,^^38^

Based on previous literature, we hypothesized that: (1) participants would perform better when spatial cues were available, (2) performance would improve when participants navigated closer to a spatial cue, (3) performance would be enhanced when the goal was located near a spatial cue, and (4) individual differences in navigational performance and reliance on spatial cues would show associations across navigation tasks and with episodic memory performance.

Given the relatively small sample size and the large number of variables included in the analyses, the present study is exploratory in nature. In particular, the dimensionality reduction approach should be interpreted as an approximation rather than a definitive model of latent processes, as such analyses can be unstable in small samples.

Nevertheless, the present results offer insights into the structure of navigation behavior. On the one hand, we observed only few strong correlations between tasks, suggesting substantial task- and context-specific influences. On the other hand, we show that a dimensionality reduction approach can be used to capture substantial shared variance across tasks. These findings provide a step toward identifying common factors underlying navigation behavior and generate hypotheses for future large-scale confirmatory studies.

## Results

### Availability of spatial cues enhances navigation performance

Across all navigation tasks, spatial cues improved navigation performance (see Figure 3, first column). In the Apple Game, participants navigated an open-field environment in which they first walked to the location of a randomly placed basket, then navigated to up to five trees to find a tree with an apple and were finally asked to return to the basket location. Half of the trials were performed without spatial cues, whereas the other half included a lighthouse landmark. Performance was significantly higher when the landmark was present, *t*(23) = 6.62, *p* < 0.001, 95% CI (43.67, 83.33). In the Island Game, participants navigated in a circular environment with distal cues and encoded the location of a randomly distributed object before returning to it either after navigating to a lure object or after being teleported to a different location, which disrupted self-motion updating. Performance was significantly reduced in the teleportation condition, *t*(23) = 9.41, *p* < 0.001, 95% CI (87.70, 137.06). In the Arena Game, participants navigated in a circular arena with distal cues and repeatedly encoded the locations of randomly distributed objects by walking to them and later retrieving their positions without the objects being present. Performance improved with increasing trial number, *t*(23) = 8.06, *p* < 0.001, *r* = 0.30, 95% CI (0.22, 0.38), indicating enhanced spatial learning with repeated exposure to environmental cues. These results show that performance was higher when spatial cues were available or could be used across tasks.

**Figure 3:**
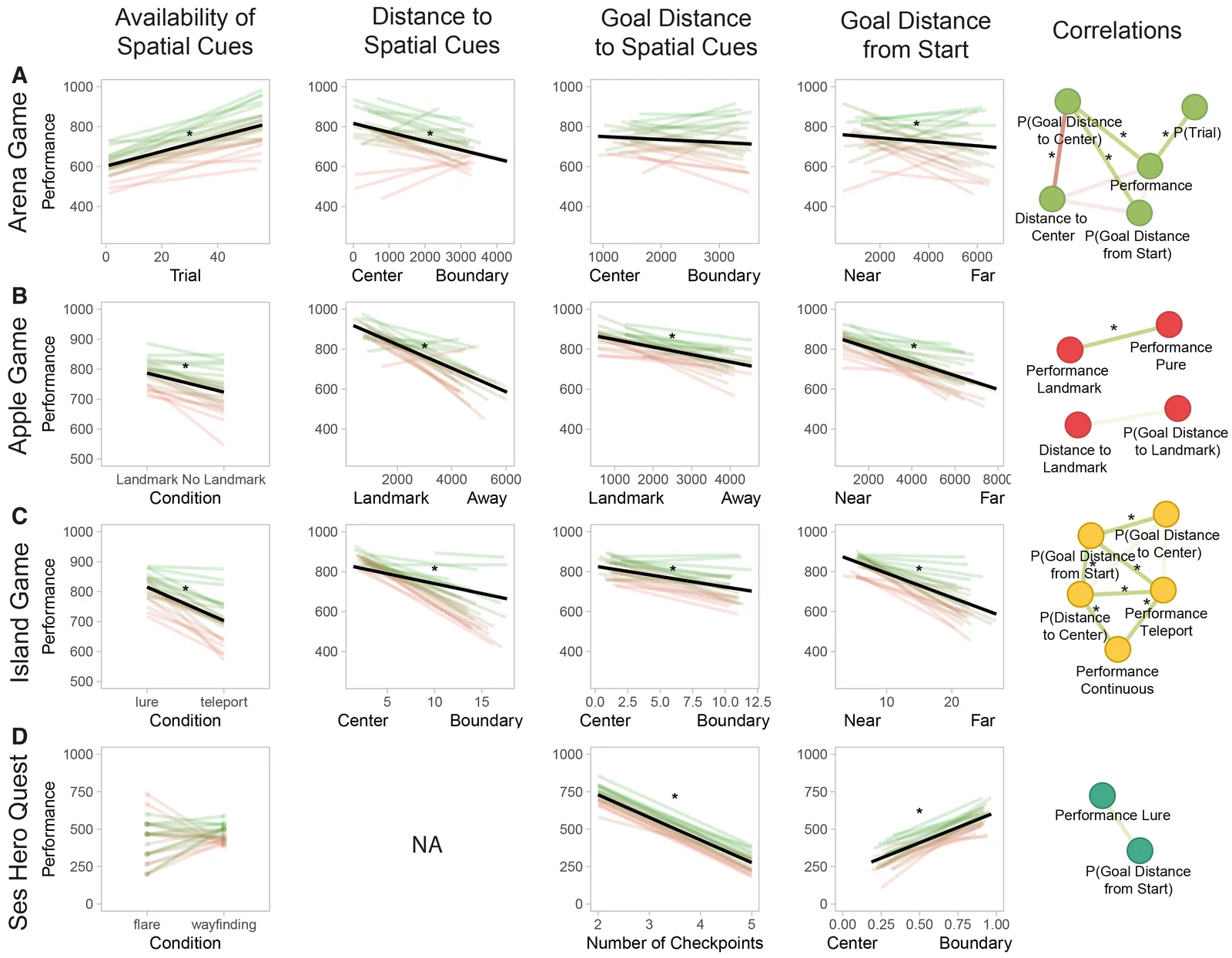
Predictors of performance across the navigation tasks Each slope in the individual plots represents a single subject’s performance, with the color gradient reflecting overall task performance, ranging from green (best performance) to orange (worst performance). (A) shows results from the Arena Game, (B) from the Apple Game, (C) from the Island Game, and (D) from the Sea Hero Quest. The plots display how performance is influenced by the availability of spatial cues (leftmost column), the average walking distance to spatial cues (second column), the goal’s distance to spatial cues (third column), and the goal’s distance from the starting location (fourth column). The rightmost column shows Spearman correlations between performance, distance to spatial cue, and the correlation values within each task that have an absolute value above 0.4. Only significant edges are displayed. P() denotes the correlation of the respective variable with task performance across subtasks. N.A. refers to not available measures; n.s., indicates not significant. Significant correlations are marked with ∗p_fdr_ < 0.05.

### Moving along spatial cues improves navigation performance

Next, we examined how movement trajectories influenced navigation performance across all tasks (see Figure 3, second column). To test this, we calculated a correlation value for each participant between the movement distance to the spatial cue in a trial and the corresponding performance. In both the Arena and the Island Games, the correlation values were positive, meaning that participants performed better in trials in which they moved closer to the center. This was significant in both the Arena Game, *t*(23) = −5.38, *p* < 0.001, *r* = −0.16, 95% CI (−0.22, −0.10), and in the Island Game, *t*(23) = −9.17, *p* < 0.001, *r* = −0.31, 95% CI (−0.38, −0.24). Similarly, in the Apple Game, performance improved when participants moved toward a landmark, *t*(23) = −6.47, *p* < 0.001, *r* = −0.29, 95% CI (−0.38, −0.20). In contrast, we found the opposite pattern in Sea Hero Quest. Sea Hero Quest is a wayfinding task in which participants study a map with a starting point and up to five checkpoints, after which the map disappears and they navigate to these checkpoints from memory. The task takes place in a bounded, maze-like environment and relies on visual scene information and optic flow during continuous movement. In this task, moving along the boundary rather than toward the center was associated with better performance, t(23) = 13.40, *p* < 0.001, r = 0.32, 95% CI (0.27, 0.37). These results show that the relationship between moving closer to spatial cues and performance differs between tasks.

### Goal location at a spatial cue improves performance

We then examined whether performance improved when the goal was located closer to a spatial cue (see Figure 3, third column). For each participant, we calculated a correlation value between the distance of the goal location to a spatial cue and performance. In the Apple Game, performance improved when the goal was nearer to a landmark, *t*(23) = −6.98, *p* < 0.001, *r* = −0.26, 95% CI (−0.34, −0.19). A similar effect emerged in the Island Game, where performance improved when the goal was nearer to the center, *t*(23) = −7.02, *p* < 0.001, *r* = −0.15, 95% CI (−0.18, −0.10). In the Arena Game, the effect was weaker but still significant, *t*(23) = −2.59, *p* = 0.016, *r* = −0.10, 95% CI (−0.19, −0.02). Together, these results suggest that performance in spatial learning and homing tasks improves when the goal is located near a spatial cue.

### Greater distance between start and goal impairs performance

Lastly, we assessed whether performance was dependent on the goal being located near the starting location. Across all tasks, we observed a significant correlation between the distance of the goal from the starting location and performance (see Figure 3, fourth column). In the Arena Game, Island Game and Apple Game, performance declined significantly as the distance from the starting location to the goal increased. Specifically, in the Island Game, the correlation was significantly smaller than zero, *t*(23) = −9.86, *p* < 0.001, *r* = −0.32, 95% CI (−0.38, −0.25). A similar effect was observed in the Apple Game, *t*(23) = −9.93, *p* < 0.001, *r* = −0.34, 95% CI (−0.41, −0.27). In the Arena Game, the correlation was also significantly smaller than zero, *t*(23) = −2.83, *p* = 0.010, *r* = −0.15, 95% CI (−0.26, −0.04). In Sea Hero Quest, performance decreased when participants had to collect more checkpoints before reaching the goal location, *t*(23) = −42.97, *p* < 0.001, *r* = −0.91, 95% CI (−0.95, −0.86). Overall, these results suggest that performance is generally better when the goal is located closer to the starting point.

### Relationships between movement preferences and reliance on spatial cues across tasks

To examine how the different metrics relate to each other, we first correlated them within each navigational task (see Figure 3, right column). For each participant, we included the average performance for each subtask and the average movement distance to the spatial cue, as well as the three correlation values from the previous steps. For the Arena Game, we additionally included the correlation between trial number and performance as an indicator of learning strength.

Several correlations survived false discovery rate (FDR) correction in the Arena Game (see Table S1 for the full correlation matrix). Participants with higher average performance showed improved performance with each additional trial. Furthermore, participants whose performance did not drop when the goal was farther away from the center did not experience a drop in performance when the goal was farther away from the starting location. Participants whose performance did not depend on the location of the goal near the spatial cue also walked more toward the center on average and had better average performance. These results suggest that some participants employed a strategy of walking toward the center, which facilitated locating the goal regardless of its initial position or proximity to the center.

In the Island Game, most pairs of variables were significantly correlated (all *p*_*fdr*_ ≤ 0.05; see Table S2 for the full correlation matrix). Performance in the continuous movement condition was significantly correlated with performance in the teleportation condition. Additionally, the better participants performed in both conditions, the less their performance depended on walking closer to the center. Conversely, participants who performed worse when walking farther from the center also performed worse when the goal was farther from the starting location. This reliance on walking to the central area was consistently associated with reduced performance in trials where the goal was farther from the center. Overall, these results demonstrate that high performers in the Island Game could complete the homing task regardless of whether they used the center for orientation or the location of the object.

In the Apple Game, only performance in the landmark condition was correlated with performance in the no-landmark condition (see Table S3 for the full correlation matrix). The average walking distance to the landmark, the relationship between walking toward the landmark and performance, and the effect of the goal location were all unrelated. These results suggest that these navigational patterns do not reflect a common process.

In Sea Hero Quest, none of the relationships were significant (*p*_*fdr*_ > 0.05; see Table S4 for the full correlation matrix). Their performance, how closely they averaged to the boundary, how dependent their performance was on the boundary, and the impact of the number of checkpoints on performance were unrelated. These findings indicate that variability in these navigational metrics does not reflect a shared underlying process within this task.

Overall, the results show that interindividual differences in various aspects of navigation behavior were correlated in two of the four navigation tasks. In both the Arena and the Island Game, high performing participants were also the ones who benefited from the object being at the center. In the other two tasks, the Apple Game and Sea Hero Quest, performing well might not require a specific navigational pattern, such as the use of spatial cues.

### Relationships between navigation tasks performance outcomes

To examine how the navigation tasks are related, we correlated the performance metrics across all the tasks (see Table S5). The strongest correlation was observed between the no-landmark condition of the Apple Game and the continuous condition of the Island Game, *r* = 0.69, *p*_*fdr*_ = 0.0027, CI (0.37, 0.85). Additionally, performance in the no-landmark condition of the Apple Game was significantly correlated with the teleportation condition of the Island Game, *r* = 0.59, *p*_*fdr*_ = 0.011, CI (0.21, 0.84). Performance in the landmark condition of the Apple Game was also significantly associated with the continuous condition of the Island Game, *r* = 0.52, *p*_*fdr*_ = 0.045, 95% CI (0.13, 0.78). No significant correlations were found between performance in the Arena Game or Sea Hero Quest and any of the other navigation tasks. These results partly reflect what would be expected based on the processes the tasks intend to assess. The no-landmark condition of the Apple Game and the continuous condition of the Island Game are both intended to assess path integration ability. However, it is somewhat unexpected that the no-landmark condition of the Apple Game relates to the teleportation condition of the Island Game, where path integration cannot be used. The fact that the Arena Game, which is supposed to assess spatial learning, and Sea Hero Quest, which is supposed to assess wayfinding, are unrelated to other tasks may indicate that the processes they tap into are largely dissociable.

Next, we expanded our analysis to include the average movement distance to the spatial cue and the correlation values derived above to examine the relationship between the tasks. Only one significant correlation was observed between the Apple Game and Island Game: participants who walked farther from the landmark in the Apple Game also did not experience a drop in performance when the goal was farther from the center in the Island Game, *r* = 0.62, *p*_*fdr*_ = 0.0155, 95% CI (0.33, 0.79) (see Figure 4). Contrary to our expectations, we did not find a relationship between walking toward the center in one task and walking toward the center in another task.

**Figure 4:**
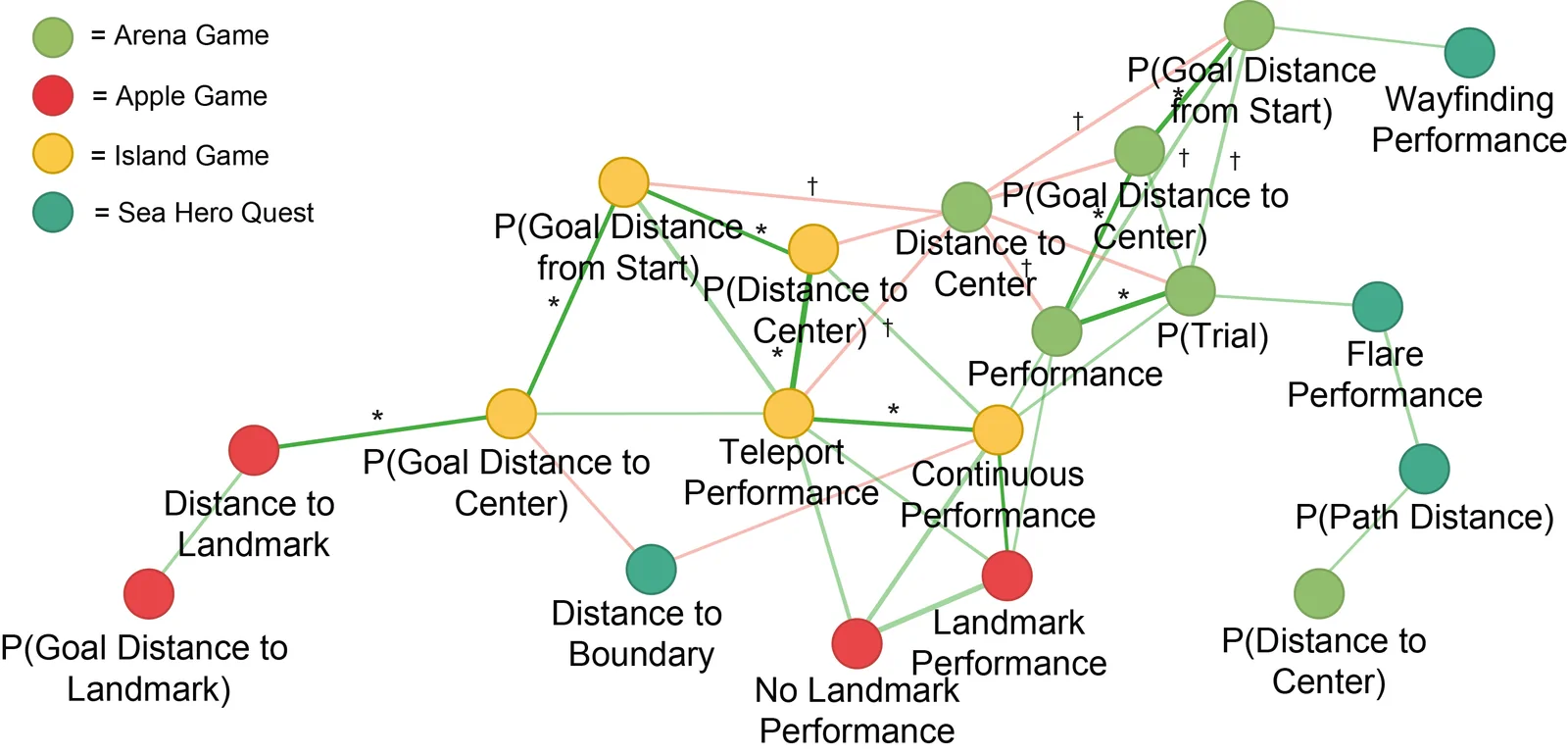
Intercorrelations of performance, movement preference, and correlation values of the four navigational tasks The network diagram illustrates the intercorrelations between performance, movement preferences, and specific task-related variables across the navigation tasks: Arena Game (green), Apple Game (red), Island Game (yellow), and Sea Hero Quest (blue). Each node represents a variable, with the color corresponding to the specific task. Only significant nodes are displayed. P() denotes the correlation of the respective variable with task performance across subtasks. Green lines indicate positive correlations, while red lines indicate negative correlations that are significant without FDR correction. The thickness of the lines reflects the strength of the correlations. Significance levels were derived from three separate multiple-comparison corrections conducted for different sets of analyses (within-task correlations, correlations between task performances, and correlations between movement preferences and performance correlations). Correlations surviving FDR correction are marked with ∗*p*_fdr_ < 0.05. ^†^*p*_fdr_ < 0.10.

### Principal component analysis reveals two distinct navigation components

To characterize the latent structure of the navigational metrics, we conducted a principal component analysis (PCA) with varimax rotation (Figure 5). The analysis yielded a two-component solution that accounted for 39% of the total variance (see Figure S1). The first component (PC1), explaining 20% of the variance, was primarily defined by spatial learning and navigation patterns in the Arena Game (e.g., distance from the center: −0.85; performance in relation to goal distance from start: 0.74). The second component (PC2), explaining 18% of the variance, was dominated by performance indices from the Apple Game and Island Game (e.g., Apple Game performance without landmarks: 0.90; Island Game continuous performance: 0.82; Table 1). To assess robustness, we additionally performed an oblique rotation (oblimin), which yielded a highly similar loading pattern. The correlation between components was small (r = 0.20), supporting the use of an orthogonal solution. We therefore retained the orthogonal varimax solution for interpretability (see Table S6).

**Figure 5:**
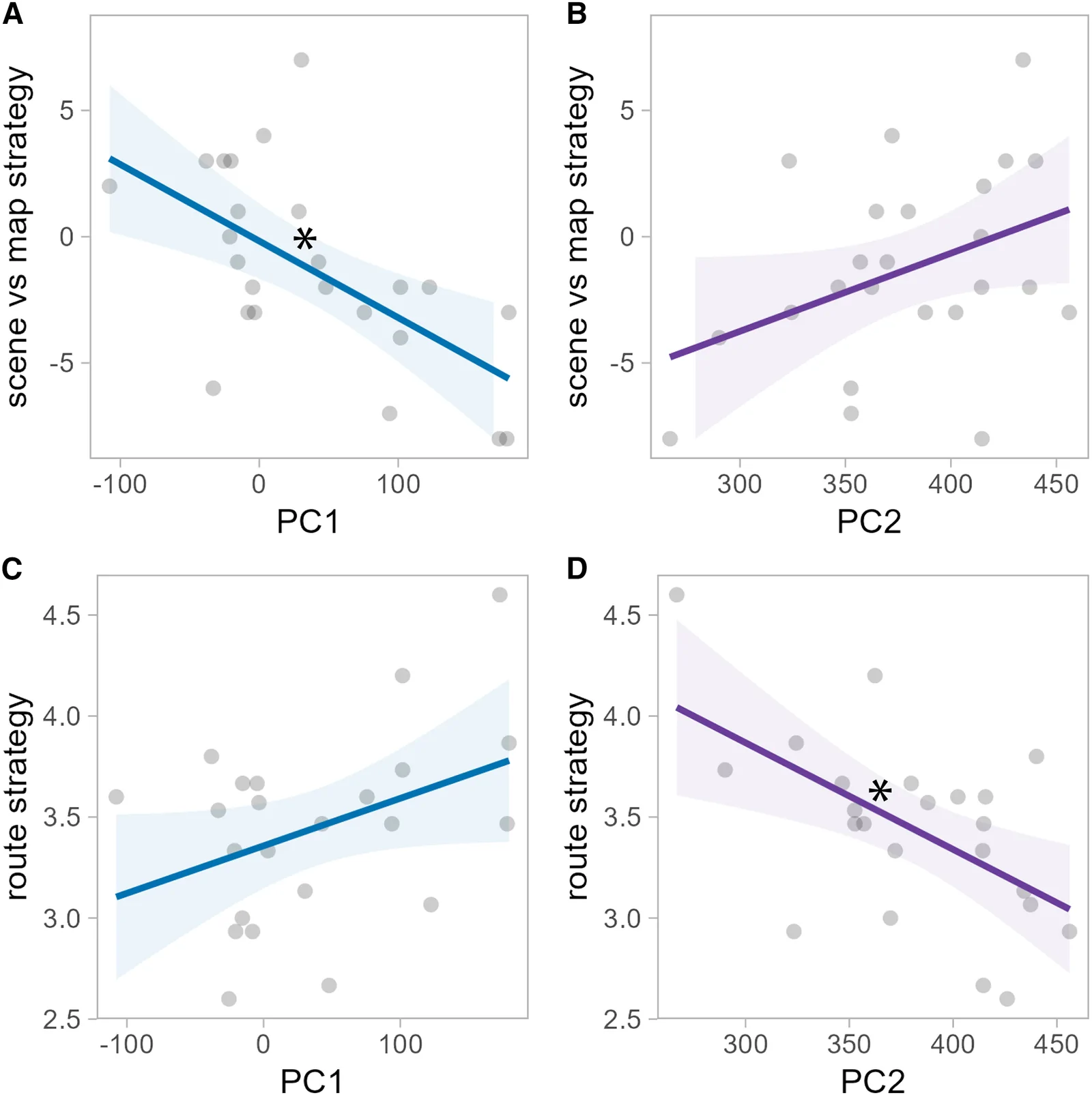
Correlation between principal components and navigational strategies The plots demonstrate the associations between principal components (PC1 and PC2) and navigational strategies (NSQ14 and NSQ44). Panels show (A) PC1 and NSQ14, (B) PC2 and NSQ14, (C) PC1 and NSQ44, and (D) PC2 and NSQ44. PC1 is negatively correlated with map-based navigation (NSQ14). Conversely, PC2 has a negative correlation with route/procedural subscale NSQ44. Regression lines with confidence intervals highlight these relationships. Significant correlations are marked with ∗p_fdr_ < 0.05.

**Table 1:** Rotated component loadings for navigation metrics

Navigational metrics	Component loading	
	1	2
Arena Game	–	–
Performance	**.68**	0.17
Distance to center	−**.85**	−.11
P (trial)	**.59**	0.30
P (distance to center)	0.15	**.42**
P (goal distance from start)	**.74**	0.05
P (goal distance to center)	**.68**	−.05
Apple Game	–	–
Performance without landmark (PPI)	−0.08	**.90**
Performance with landmark (LPI)	−0.01	**.76**
Distance to landmark	**.59**	−.28
P (goal distance to landmark)	**.47**	−.16
P (goal distance from start)	0.13	0.12
P (distance to landmark)	0.08	0.07
Island Game	–	–
Performance (continuous)	0.21	**.82**
Performance (teleport)	**.55**	**.68**
Distance to center	**.54**	0.16
P (distance to center)	**.49**	**.42**
P (goal distance to center)	**.49**	**.52**
P (goal distance from start)	**.59**	**.42**
Sea Hero Quest	–	–
Performance wayfinding	−0.26	0.13
Performance flare	0.02	**.39**
Near boundary	0.02	−**.60**
P (near boundary)	0.07	**−.32**
P (path distance)	−.23	0.21

Loadings are from a principal component analysis on the correlation matrix with varimax rotation. Loadings with absolute values ≥ .30 are shown in bold.

We then used the components derived from PCA to relate them to the navigational strategy questionnaires (NSQ14: α = 0.60; NSQ44: α = 0.80; see Table S7). Two significant associations emerged: PC1 was negatively correlated with NSQ14, *r* = −0.60, *p* = 0.0019, 95% CI (−0.80, −0.26), indicating that participants reported less map-based navigation and instead tended to picture scenes along the route. PC2 was negatively correlated with the route/procedural subscale of NSQ44, *r* = −0.56, *p* = 0.0053, 95% CI (−0.79, −0.20), indicating that participants reported relying less on remembering routes as sequences of landmarks and turns.

Taken together, these findings suggest that the observed behavioral pattern can be captured by two components reflecting distinct navigational processes. The first component reflects a preference for walking toward the center and performing similarly regardless of whether the goal is located near a spatial cue, and it was associated with a more scene-based navigation strategy. The second component reflects path integration ability and was associated with reduced reliance on remembering the route as a sequence of route segments and turning points.

### Effects of event boundaries on memory

In the episodic memory tasks, we investigated whether event boundaries modulate episodic memory (see Figure S2). Specifically, the auditory event boundary task examined the impact of auditory boundaries on the temporal structure of memory. The presence of boundaries did not lead to a significant subjective time expansion effect, t(23) = −0.60, *p* = 0.553, *d* = −0.12, 95% CI (−0.10, 0.05). Similarly, temporal order memory did not differ significantly between boundary-spanning and same-context pairs, *t*(23) = 1.30, *p* = 0.207, *d* = 0.27, 95% CI (−0.02, 0.08). However, temporal order memory was significantly above chance level in both the same-context condition, *t*(23) = 3.05, *p* = 0.006, 95% CI (0.53, 0.66), and the boundary-spanning condition, *t*(23) = 3.75, *p* = 0.001, 95% CI [0.53, 0.60]. Source memory for the tone/ear associated with each item was significantly better for items immediately following a boundary than for items within the same context, *t*(23) = −2.74, *p* = 0.012, *d* = 0.56, 95% CI (0.01, 0.09). In this task, we therefore replicated one of three boundary effects previously found.

We also examined the modulation of memory by event boundaries in the movies. For the Birdman task, temporal order accuracy was significantly reduced for image pairs that contained an event boundary compared to pairs without a boundary, *t*(23) = 4.10, *p* < 0.001, *d* = 0.84, 95% CI (0.07, 0.23). However, this effect was not observed in the Sherlock task, *t*(23) = −0.03, *p* = 0.553, *d* = −0.12, 95% CI (−0.12, 0.06). The movies demonstrated a boundary effect on temporal compression in memory. Participants took more time to recall events between two images when boundaries were present, resulting in reduced temporal compression. This was observed in both the Birdman task, *t*(23) = 2.46, *p* = 0.019, *d* = 1.35, 95% CI (0.21, 2.49), and the Sherlock task, *t*(23) = 2.35, *p* = 0.028, *d* = 0.48, 95% CI (0.11, 1.75). However, event boundaries did not significantly affect distance estimation in either the Birdman movie, *t*(22) = −1.48, *p* = 0.153, *d* = −0.31, 95% CI (−36.1, 6.02), or the Sherlock movie, *t*(23) = 1.47, *p* = 0.154, *d* = 0.30, 95% CI (−3.83, 22.8). Thus, we found an impact of event boundaries on some aspects of episodic memory with naturalistic stimuli.

In the Bus Task, we examined how turns affect recognition memory. Contrary to previous findings, there was no significant increase in “re-experience” responses at pre-turn intersections, *t*(23) = −1.09, *p* = 0.288, *d* = −0.22, 95% CI (−0.1, 0.3). This pattern remained unchanged when considering only correctly recognized familiar intersections, *t*(23) = −1.09, *p* = 0.288, *d* = −0.22, 95% CI (−0.1, 0.03). Additionally, no significant effect was observed on the temporal compression index, *t*(21) = −0.05, *p* = 0.959, *d* = −0.01, 95% CI (−0.19, 0.18). We were therefore unable to detect boundary-related effects in this task.

### Relationships within and between event segmentation tasks

Before examining whether navigation tasks are related to episodic memory tasks, we tested whether the episodic memory metrics were correlated within and between the episodic memory tasks. In our analysis, we included average performance in the different subtasks and boundary effects within each task. Surprisingly, most metrics were not related, even within the same task. Only one significant correlation was found in the auditory event boundary task (see Tables S8–S11). Specifically, the reduction in temporal order memory for boundary-spanning pairs was more pronounced in participants who generally had higher overall order memory performance, *r* = −0.71, *p*_*fdr*_ = 0.003, 95% CI (−0.87, −0.40).

When correlating the metrics between tasks, some significant correlations appeared. The compression index in both the Sherlock and Birdman tasks was significantly correlated, *r* = 0.68, *p*_fdr_ = 0.023, 95% CI (0.344, 0.88). Additionally, the data revealed that participants who estimated longer distances in the Birdman task also had a greater reduction in distance estimation in the Sherlock task when boundaries were present, *r* = −0.76, *p*_fdr_ < 0.01, 95% CI (−0.88, −0.53).

To explore the underlying structure of these variables, we conducted a PCA. The results of the parallel analysis suggested retaining a single component, which accounted for 21% of the total variance (see Figure S3). This component was mainly associated with distance estimation in the Birdman task and the effect of boundaries on distance estimation in the Sherlock task (e.g., Birdman task distance estimation, 0.80; Sherlock task boundary effect on distance estimation, 0.80).

### Relationships between navigation and episodic memory tasks

Finally, we tested for any correlation between spatial navigation and episodic memory tasks. No significant correlations were found, suggesting that there is no evidence of a relationship between the two domains in this sample.

## Discussion

This study integrates experimental and correlational approaches to examine shared and distinct processes underlying navigation behavior across multiple virtual navigation tasks. Although our findings are limited in their generalizability due to the small sample size, several consistent patterns emerged that can inform future research. First, spatial cues reliably enhanced navigation performance across different task demands. Second, we observed cross-task correlations, as well as many non-significant relationships, suggesting that navigation behavior is shaped by shared and task-specific processes, as well as context-dependent influences. Third, PCA can be used to extract components from navigational metrics that are linked to distinct self-reported navigational strategies.

A key finding is that participants strategically use spatial cues to enhance navigation performance across different task demands. Performance improved when spatial cues were present, as in the Apple Game when landmarks were present, or in the Island Game, where participants could use both distal cues and visual flow to navigate. Goals were better remembered when they are located closer to spatial cues. In the Arena Game and Island Game, both of which have a circular arena, walking toward the center improved performance. One possible explanation is that the center provides a better view of the surrounding area. This might facilitate route-based navigation, in which participants look for a specific snapshot of distal cues that can be followed as a sequence of additional movements or turns to reach the goal. Alternatively, going toward the center may help participants reconstruct a cognitive map of the environment that can be used to find the goal location. These results align with previous findings that spatial cues facilitate successive navigation by providing reliable orientation markers.^12^^,^^39^ This implies that if virtual navigation tasks aimed at assessing path integration ability include spatial cues, the ability to strategically use those cues will always confound the measurements.

One intriguing aspect of our findings is the variability in how much people rely on spatial cues. For instance, in the Arena Game, a weak group-level effect of goal distance from the center on performance was observed. However, the extent to which performance depended on the proximity of the goal to the center was still correlated with other variables. Specifically, participants whose performance did not drop when the goal was farther away from the center tended to perform better on average, experience a smaller drop when the goal was farther away from the starting location, and spend more time at the center. A similar relationship was found in the Island Game. Participants whose performance did not drop when the goal was farther away from the center also did not rely on the goal being near the starting location. Therefore, even if there is no strong group-level effect, the variance in correlation values may provide meaningful information about how much participants rely on spatial cues.

Although comparable findings emerged between the tasks, correlations between them were limited. Notably, two significant correlations emerged between the Apple Game and the Island Game. First, performance in the no-landmark condition of the Apple Game was associated with performance in the continuous condition of the Island Game. These variables may therefore capture the ability for path integration. Second, participants who walked farther from the landmark in the Apple Game did not experience a drop in performance when the goal was far from the center in the Island Game. However, it is surprising that most movement preferences and reliance on spatial cues did not significantly correlate across tasks. For example, walking toward the center in one task was not related to walking toward the center in the other task. Notably, previous studies investigated participants with the APOE e4 gene, which is a risk factor for Alzheimer’s disease. These participants showed reduced central pref.^11^, increased reliance on a landmark in the Apple Game^12^ and impaired wayfinding performance.^40^ These variables were not significantly correlated in the present study. These discrepancies raise critical questions about the reliability and validity of metrics derived from virtual navigation tasks.

There are three potential explanations for the limited cross-task correlations: (a) the tasks may not assess navigational processes, but rather, contextual factors or states, (b) the tasks may reveal group-level differences, with a large enough sample size, but this is not reliable enough to predict individual behavior, and (c) the tasks may not measure shared processes.

Regarding (a), navigation behavior may be highly context dependent or state dependent. Navigation patterns might not reflect stable traits but rather adaptive responses to specific contexts based on the layout and cue distribution of each environment.^18^ Factors such as stress, upbringing, task familiarity, computer gaming experience, and even sleep contribute to the observed navigation performance.^14^^,^^21^^,^^22^^,^^23^^,^^41^ In our study, familiarity with using a joystick might add to the variance in performance. Moreover, because testing lasted several hours, participants may have experienced varying degrees of fatigue, stress or sleepiness across the sessions, potentially affecting different tasks to different extents.

In (b), the relatively small sample size may have limited statistical power. A sensitivity power analysis indicated that, with a sample size of *n* = 24, the study was sufficiently powered to detect only relatively large correlations (approximately *r* = 0.54 for 80% power and *r* = 0.60 for 90% power at α = 0.05). This implies that small to moderate associations may have gone undetected. It also remains an open question whether the metrics derived from these navigation tasks reliably reflect underlying navigation processes. One way to assess reliability is through test-retest designs, which have been conducted for the Sea Hero Quest task.^42^^,^^43^ To our knowledge, comparable reliability assessments are not yet available for the other tasks used here. Nevertheless, the individual tasks replicated previously reported effects, for example improved performance in the presence of landmarks in the Apple Game and learning effects across trials in the Arena Game, suggesting that they are capable of reliably detecting task-specific effects. However, these effects may be too small to reveal consistent relationships across multiple variables in a sample of this size.

Regarding (c), the limited correlations raise the possibility that different navigation tasks engage in largely independent processes. The tasks used in the present study vary substantially in their task demands and environmental structure. For example, the Arena Game primarily requires remembering object locations within a bounded environment and is designed as an allocentric spatial learning task inspired by the Morris Water Maze, whereas tasks such as the Apple Game involve returning to a starting location, placing greater demands on path integration and working memory abilities. In contrast, Sea Hero Quest involves navigation through maze-like environments following map viewing rather than remembering positions within circular arenas, which may place greater demands on the transformation from allocentric to egocentric reference frames and on route-based navigation processes. To examine potential shared processes across these tasks, we conducted a PCA. This analysis identified two components that together accounted for 39% of the variance, suggesting that while some common processes may be captured, a substantial proportion of variance remains task specific.

The extracted components showed significant associations with self-reported navigational strategies, providing further support for the validity of the PCA solution. Greater contribution to the first component was associated with increased self-reported use of a scene-based strategy, in which routes are planned by recalling visual scenes encountered along the way rather than by constructing a map. The strongest contributor to this component was a tendency to navigate closer to the center of the arena on average and showing comparable performance regardless of whether the goal was located farther from the center or the starting location. This component also included metrics from the Island Game indicating that participants did not rely on the goal being near the center or the starting location. This component may capture a specific strategy in which participants remember scenes along a route to effectively locate a goal, even when it is not situated in a salient location. The second component was primarily defined by performance in the Apple Game in trials without a landmark and by performance in the Island Game under continuous navigation, both of which are designed to assess path integration ability. Greater contribution to this component was associated with a lower tendency to report using routes or landmarks for orientation, suggesting that individuals who rely less on landmarks may navigate more effectively in environments lacking external spatial cues. Because the two components were orthogonal, the scene-based strategy captured by the first component appears to be dissociable from the ability to perform path integration in the absence of landmarks.

Because of the parallels described in the literature, we also explored possible relationships between navigation and episodic memory. However, we had difficulty replicating the effects of event boundaries in episodic tasks. In the auditory event boundary task, we only observed an effect on source memory, while effects on order or perceived temporal distance were absent. In the Bus Game, the effect of turns on recognition memory was not evident in our data. In the movie tasks, boundaries affected temporal compression and order in only one of the films. Furthermore, we did not observe consistent relationships between metrics either within or across tasks, for both overall performance measures and boundary-related effects. While some variables may reflect distinct underlying constructs, one might nevertheless expect at least moderate correlations for conceptually similar measures (e.g., temporal order memory) across tasks. At present, it remains unclear to what extent these measures are reliable. Notably, previous work examining correlations across multiple episodic memory tasks has also reported low correlations.^44^ Taken together, this raises the possibility that current measures lack sufficient reliability. Therefore, the absence of correlations between episodic and spatial measures should not be interpreted as strong evidence for their independence.

### Limitations of the study

Some limitations should be mentioned. This study was designed as an extensive investigation to assess the feasibility of combining multiple virtual navigation and episodic memory tasks within a single cohort. As such, the sample size represents a limitation that constrains the generalizability of the inter-individual correlation analyses. Consequently, the absence of significant correlations between certain navigation tasks should be interpreted with caution. In addition, the sample size limits the ability to draw conclusions about the underlying latent structure of the observed relationships. PCA was employed as a dimensionality reduction technique rather than a method for identifying underlying processes. To more rigorously characterize shared processes, larger samples and more sophisticated approaches such as factor analysis with model fit indices would be required. Therefore, the identified components should be considered exploratory.

Furthermore, it is important to note that both the selection of tasks and the sample constrain the processes that can be detected. The current task battery was chosen because these tasks have previously been shown to reveal altered behavioral patterns in individuals at genetic risk for Alzheimer’s disease. As such, the identified components likely reflect the specific cognitive demands captured by these tasks rather than a comprehensive taxonomy of navigation processes.

In addition, animal and human research suggests that navigation depends on both self-motion-based updating and the use of external spatial cues such as landmarks and boundaries.^1^^,^^2^^,^^3^ In the present study, however, the virtual tasks were administered on desktop or mobile devices and therefore provided little to no genuine idiothetic input, while relying predominantly on allothetic visual information such as optic flow, landmarks, and environmental geometry. As a result, the ability to use idiothetic self-motion cues cannot be directly captured by these tasks.

Finally, the present sample was strongly imbalanced with respect to sex (21 women, 3 men), which further limits the generalizability of our findings. Previous research has consistently reported sex differences in spatial navigation, with males on average outperforming females, particularly in recall-based tasks,^45^ with some evidence indicating that the male advantage is more pronounced when using an allocentric perspective compared to an egocentric perspective.^46^ If such differences in underlying cognitive processes are present, they may also influence the pattern of correlations observed between navigation measures. Accordingly, it is unclear whether and to what extent these results are generalizable.

Methodological factors may also have obscured the relationships between tasks. For example, the order of trials in the Apple and Arena Games was randomized, potentially introducing additional variability. Moreover, the extensive testing protocol, spanning approximately 15 h over three days, may have contributed to participant fatigue or training effects that carried over across tasks, thereby influencing performance and increasing variability. In addition, Sea Hero Quest was completed at home, which may have introduced additional uncontrolled factors, such as distractions. Future studies should focus on developing tasks that are not only reliable and valid but also efficient. Creating shorter, more focused tests that reliably measure constructs of interest while minimizing fatigue will be essential. Optimizing task design by removing levels that compromise internal consistency, incorporating test-retest reliability assessments, and establishing external validity through correlations with real-world behavior will be critical.

Future research should, beyond employing more sophisticated latent variable approaches, also investigate brain-behavior associations within functional and structural brain networks to better characterize the nature of the extracted factors. Such analyses could help disentangle whether the shared variance reflects stable inter-individual differences or is driven by task- and context-specific influences. Ultimately, identifying these latent navigational factors and comparing them across groups may help clarify, which cognitive processes are selectively affected in aging and in individuals at increased risk for Alzheimer’s disease.

### Conclusion

In summary, our study provides a crucial step toward understanding the complexity of spatial navigation. By employing multiple tasks and integrating both performance and reliance on spatial cues, we found that navigational behavior may be more context and trait specific than previously thought. Although cross-task correlations were limited, PCA revealed distinct behavioral components, suggesting that different navigational processes can be dissociated. These findings serve as a proof of principle that a data-driven taxonomy of spatial navigation behavior is feasible. Overall, our study paves the way for a more thorough evaluation of the underlying processes that drive navigational behavior.

## Resource availability

### Lead contact

Further information and requests for resources should be directed to and will be fulfilled by the lead contact Varnan Chandreswaran (varnan.chandreswaran@rub.de).

### Materials availability

This study did not generate new unique reagents.

### Data and code availability

• Data reported in this paper are available on the Open Science Framework (OSF) at https://doi.org/10.17605/OSF.IO/2YG4E (accession code: 2yg4e).
• Analysis scripts used to generate the reported results are available on the Open Science Framework (OSF) at https://doi.org/10.17605/OSF.IO/2YG4E (accession code: 2yg4e).
• Any additional information required to reanalyze the data reported in this paper is available from the lead contact upon request.

## Acknowledgments

We acknowledge funding via DFG grant project no 429281110. D.S. is currently a Research Associate at the Fonds de la Recherche Scientifique (F.R.S.-FNRS).

## Author contributions

Conceptualization, V.C., N.A., and H.Z.; data curation, V.C. and D.P.; formal analysis, V.C.; funding acquisition, N.A. and H.Z.; investigation, V.C. and D.P.; methodology, V.C., D.S., A.B., D.C., I.B., G.C., L.D., H.S., M.H., M.S., L.F., M.B., J.O., L.K., N.A., and H.Z.; resources, D.S., A.B., D.C., I.B., G.C., L.D., H.S., M.H., M.S., L.F., M.B., J.O., and L.K.; software, V.C. and A.B.; project administration, V.C., N.A., and H.Z.; supervision, N.A. and H.Z.; validation, V.C., N.A., and H.Z.; writing – original draft, V.C.; writing – review and editing, V.C., D.S., A.B., D.C., I.B., G.C., L.D., H.S., M.H., M.S., L.F., M.B., J.O., L.K., D.P., N.A., and H.Z.

## Declaration of interests

The authors declare no competing interests.

## STAR★Methods

### Key resources table

**Table:** 

REAGENT or RESOURCE	SOURCE	IDENTIFIER
**Deposited data**		

Data for the analysis of this paper	OSF	Accession code: 2yg4e; https://doi.org/10.17605/OSF.IO/2YG4E

**Software and algorithms**		

R 4.4.1	CRAN	https://www.r-project.org/
MATLAB (2021b)	MathWorks	https://www.mathworks.com/products/matlab.html

### Experimental model and study participant details

Twenty-four adult human participants were recruited through advertisements at Ruhr University Bochum. Of these, 21 were women and three were men, ranging in age from 18 to 34 years old (mean age = 21.84 years). They participated in return for course credit or a €10 expense allowance per hour. All participants completed the same study protocol; there was no allocation to between-participant experimental groups, and task conditions were administered within participants. Written informed consent was obtained from all participants in accordance with ethical standards approved by the Ethical Committee of the Faculty of Psychology at Ruhr University Bochum (approval number: 560), and consistent with the Declaration of Helsinki. Participants were excluded if they: (1) were under 18 years old; (2) had perception difficulties (e.g., impaired vision or hearing); (3) had mental health issues; (4) did not speak German; (5) had watched the first episode of BBC Sherlock or the film Birdman; or (6) had experienced problems (e.g., dizziness) while performing virtual navigation tasks. Because the sample included only three men, sex-related associations and sex-by-condition effects could not be estimated reliably and were not tested.

### Method details

#### Experimental tasks

Participants completed eight distinct tasks, including four navigation tasks within a virtual environment: a spatial learning task “Arena Game”,^11^ a path integration task with or without different spatial cues “Apple Game”,^12^ a path integration task with or without self-motion cues “Island Game”^37^ and a wayfinding task “Sea Hero Quest”.^8^ An overview of the navigation tasks is provided in Figure 1. In addition, participants completed four event segmentation tasks: a sequential learning task with auditory event boundary “Auditory Event Boundary Task”,^32^ a recognition memory task with spatial turn “Bus Game”,^34^ and two event segmentation tasks with movies “Sherlock Task” and “Birdman Task”.

##### Spatial learning, “arena game”

This task was designed to test memory for the location of objects within a circular virtual arena featuring mountains as spatial cues. At the beginning of each trial, participants were shown an image of an object and asked to navigate to its correct location within the arena. After making a choice, the correct location was revealed, and participants must then move to that position. Initially, participants had to guess, but as the same objects are repeated, they can use the spatial cues to remember the correct locations. Each participant completes 56 trials in total, learning the positions of 8 different objects. The position of the participant was logged every 0.1 s (10 Hz). This task was developed in Unreal Engine. Similar versions of this task have been used in previous studies.^9^^,^^10^^,^^11^

##### Homing “apple game”

This task assesses path integration abilities in a virtual environment with and without the help of spatial cues. The game takes place in an open, grassy field. Each trial consists of three phases: First, participants memorized the location of a basket. Then, they navigated toward a tree bearing a red apple. Finally, they attempted to return to the basket’s location. Participants received performance feedback based on how accurately they returned to the basket. The task was completed under two conditions: with and without a landmark (lighthouse) serving as spatial references. Participants performed 64 trials. The position of the participant was logged every 0.2 s (5 Hz). This task was developed using Unreal Engine and has been used in several studies.^12^^,^^13^^,^^14^

##### Homing “Island game”

This task assesses homing behavior in a virtual island in both a continuous navigation condition and a teleportation condition. In the continuous navigation condition, participants traversed the virtual environment to reach a goal location. After reaching the goal, they navigated to a different starting point, indicated by a white cube, and then had to find their way back to the previously collected goal location. In the teleportation condition, participants were relocated instantly to a new starting point after collecting the goal image. From this new location, they had to navigate back to the initial goal location. Although we did not include it in our analysis, the task also assesses item memory, in which goal and lure images are displayed on the screen and participants use the left and right arrow buttons to select the goal image. In total participants completed 135 trials. The position of the participant was logged approximately every 0.03 s (approximately 30–35 Hz). This task was adapted from Pink et al.^37^ and implemented in Panda3D.

##### Wayfinding “sea hero quest”

This smartphone task required participants to use wayfinding to navigate a boat to find checkpoints, using a map to help them complete the task. Participants began by studying a map displaying their starting location and several sequential checkpoints represented by buoys with flags indicating their order. After a set time to study the map, participants had to find the checkpoints in order, all while being timed. While navigating their boat through the virtual environment, participants had to maintain awareness of their location using self-motion and spatial cues. The number of checkpoints that participants needed to collect before completing a level varied. Some levels included fog that obscured their view, while others did not. The levels included in this study were: 1, 2, 3, 6, 7, 8, 11, 12, 13, 16, 17, 18, 21, 22, 23, 26, 27, 28, 31, 32, 33, 36, 37, 38, 41, 42, 43, 46, 47, 48, 51, 52, 53, 56, 57, 58, 61, 62, 63, 66, 67, 68, 71, 72 and 73. The position of the participant was logged every 0.5 s (approximately 2 Hz). This task was adapted from Coutrot et al.^8^

We also included four event segmentation tasks that varied in the memory functions assessed, the type of stimuli used, and the way event boundaries were manipulated.

##### Episodic memory “auditory event boundary task”

This task assesses the influence of auditory event boundaries on temporal and source memory. Participants viewed a sequence of 32 greyscale images, each displayed for 2.5 s, followed by a 3-s gap. During these gaps, a tone was played in either the left or right ear. The ear in which the tone was played determined which hand the participants used to categorize the object as being found indoors or outdoors. The tone/ear pairing remained consistent for the first eight images, establishing a stable pattern. After eight images, the tone switched ear and pitch, marking a new ‘event’. This pattern was repeated throughout the sequence. Participants completed two memory assessments. In the temporal memory test, they were shown pairs of items from the earlier sequence and asked to identify which item had appeared first. They were then asked to rate how close together or far apart the two items were in time. The pairs included items from within the same auditory event, as well as pairs that crossed an event boundary. In the source memory test, participants were shown individual items and asked to recall whether the item had been presented in the left or right ear. The items were presented in a randomized order and there was no time limit for responses. The task was adapted from that of Clewett et al.^32^ and implemented in E-Prime Version 2.0.

##### Episodic memory “bus game”

This task assesses the impact of navigational turns on recognition memory and temporal compression. Participants navigated the streets of Chicago, simulating the training of a bus driver learning a new route. They were briefed on the task of simulating bus driver training on a new Chicago route, with a focus on bus stop intersections marked with red bars. They were instructed to observe the scenery and 12 bus stops: Six were located before turns (pre-turn intersection) and six were located along straight segments (mid-segment intersection), all at potential turn intersections. Stop durations varied from 1 to 12 s, with each bus stop occurring directly before a turn. After completing the task, participants performed a recognition memory task to assess their ability to distinguish between the twelve familiar bus stops and twelve novel intersections, which were presented in a random order. For each image, participants indicated whether they had experienced the waiting event before (recollection), recognized it without recalling any associated context (familiarity), or thought it was new. ‘Re-experience’ responses captured detailed recollections of the stops, while ‘know’ responses indicated a sense of familiarity. Additionally, participants completed an assessment in which participants were shown pairs of images and asked to mentally replay everything that occurred between the two intersections, with the duration of the replay being measured. This task was adopted from Brunec et al.^34^

##### Naturalistic episodic memory “sherlock task” and “birdman task”

Two event segmentation tasks were developed to assess the effect of episodic boundaries on recognition and temporal memory. Both tasks involved watching a film: Sherlock, a BBC series (48 min), and Birdman, a single-shot film (the first 52 min). After watching each film, participants completed three memory tasks related to its content. These tasks were organized into two distinct blocks.

In the first block, participants were shown 16 pairs of images, each consisting of individual frames from the respective film. The image pairs were carefully selected to vary in the number of event boundaries between them. Specifically, half of the pairs were derived from segments with minimal event boundaries, while the other half were from segments with a high number of event boundaries. Event boundaries were determined by following the procedure from Baldassano et al.^47^ Additionally, the trials were designed to differ in temporal distance: half of the image pairs were separated by 45 s and half by 75 s. These two manipulations (high vs. low boundaries and 45 vs. 75 s) were balanced across the trials. Upon viewing each pair of images, participants were asked to mentally replay all the events that occurred between them. They were then required to rate the perceived temporal distance between the two images by typing in the time.

The second block involved assessing temporal order memory. Participants were shown pairs of images from the film separated by 10 s. They were then asked to judge whether the images were presented in the correct chronological order. The trials were equally divided between pairs of images containing an event boundary and pairs without. The memory assessment was adapted from^38^ and implemented using PsychoPy.

##### Navigational strategies questionnaires

In addition to completing the experimental tasks, participants responded to two questionnaires designed to assess their self-reported navigational strategies. The first was the 44-item “Navigation Strategy Questionnaire” (NSQ44^16^), which measures individual differences across three key navigation dimensions: egocentric spatial updating, survey-based strategies, and procedural/route-based strategies. Participants rated each item on a 5-point Likert scale, ranging from 1 (totally disagree) to 5 (totally agree). Additionally, participants completed a shorter 14-item questionnaire created to evaluate their tendency toward map-based navigation (NSQ14^15^). Each item presented two response options, one reflecting a map-based strategy and the other a scene-based strategy. A mapping tendency score was calculated by determining the difference between the number of map-based and scene-based responses.

#### Experimental procedure

Testing took place over three days following a fixed schedule (see Figure 2). On the first day, participants arrived at the laboratory, provided written informed consent, completed neuropsychological testing, and performed the Apple Game, Bus Game, Auditory Event Boundary Task, and Birdman Task. On the second day, participants completed selected levels of Sea Hero Quest at home and filled out the questionnaires. On the third day, they returned to the laboratory to complete the Arena Game, Island Game, and Sherlock Task. Upon completion of the study, participants received compensation.

#### Behavioral data

##### Data analysis

A total of 24 participants took part in the study. For each navigation task, we derived trial-wise measures of performance, movement preference, and goal location. In the Apple, Arena, and Island Games, raw navigation accuracy was quantified as drop error, defined as the Euclidean distance between the true goal location and the participant’s response location. For ease of interpretation, we transformed drop error into a performance score such that higher values indicate better performance. Specifically, within each task we linearly inverted drop error using the task-wide minimum and maximum observed across all participants and trials and scaled the resulting values to an approximately 0–1000 range.

Movement preference was quantified as the mean *distance to the spatial cue* within each trial, computed by averaging the Euclidean distance between every logged position sample and the spatial cue (either the center or the landmark). Similarly, *goal distance to the spatial cue* was quantified as the Euclidean distance between the goal location and the spatial cue. We also computed the Euclidean *distance between the goal location and the starting location*. The starting location was defined as the position at trial onset in the Arena Game, the location of the lure or the relocation position in the Island Game, and the position at the final tree (apple location) in the Apple Game.

In Sea Hero Quest, *performance* was quantified as the total distance traveled by the participant, with lower distances indicating better performance. To maintain consistency with the other navigation tasks, this measure was inverted and scaled in the same manner as described above, such that higher values indicate better performance. *Distance to the boundary* was quantified as the proportion of time points at which the participant was positioned closer to the nearest boundary than the center of the environment. *Goal distance from the starting location* was indexed by the number of checkpoints that participants were required to collect to complete a level.

In the event segmentation tasks, we assessed various aspects of memory, such as temporal and recognition memory. Temporal *order accuracy* in the Auditory Event Boundary Task, as well as in the Sherlock and Birdman Tasks, was assessed by calculating the average number of correct responses. Temporal distance in the Auditory Event Boundary Task was calculated by averaging the response values ranging from 1 (very close) to 4 (very far). For both films, *temporal distance* was calculated based on the time the participants typed in. The *temporal compression* index for the films and the Bus Game was calculated as the ratio of the time taken to mentally re-experience the segment to its actual duration. *Source memory* in the Auditory Event Boundary Task was the proportion of correctly remembered ear/sound side. Recognition memory in the Bus Game was the proportion of ‘re-experience’ responses.

For the virtual navigation tasks, MATLAB (2021b, The MathWorks Inc., Massachusetts, US) was used to analyze log files. Statistical analyses were conducted using R.^48^

#### Statistical analysis

We adopted a consistent statistical framework to examine the influence of spatial cues on navigation performance and the impact of event boundaries on memory performance across tasks.

##### Analysis of spatial navigation tasks

For the spatial navigation tasks, we first examined the role of spatial cue availability on navigation performance. In the Apple Game and Island Game, performance was compared across conditions using paired two-tailed t-tests. Specifically, in the Apple Game, performance was contrasted between trials with and without landmarks, whereas in the Island Game, performance was compared between continuous navigation and teleportation conditions. In the Arena Game, Spearman’s correlation coefficients were computed for each participant to assess the relationship between trial number and performance, indexing learning across trials. These individual correlation coefficients were Fisher z-transformed and then tested against zero using one-sample two-tailed t-tests.

Next, we quantified the influence of movement preferences and goal location on performance. For each participant, correlation coefficients were calculated between trial-wise performance and (i) distance traveled relative to spatial cues, (ii) distance between the goal location and the spatial cue, and (iii) distance from the starting location to the goal. These correlation coefficients were subsequently Fisher z-transformed and tested against zero using one-sample two-tailed t-tests.

##### Analysis of event segmentation tasks

For the event segmentation tasks, we tested the effect of event boundaries on memory performance using paired two-tailed t-tests. We compared memory performance in terms of order memory, temporal compression, and perceived temporal distance for item pairs that contained event boundaries versus those that did not. In the Auditory Event Boundary Task, we also compared source memory for items immediately following a tone switch (indicating an event boundary) against other items within the same context, excluding the first and last items. For the Bus Game, we compared recognition accuracy for intersections located just before a turn versus those located along straight segments.

##### Analysis of intercorrelations

To examine potential relationships across the different tasks, we computed Spearman’s correlation coefficients for the variables described above. We investigated correlations within the navigation tasks, within the episodic memory tasks, and between spatial and episodic measures. To control for multiple comparisons, false discovery rate (FDR) corrections were applied separately to the within-task and between-task correlation analyses.

Additionally, we conducted a PCA on both the navigation and episodic memory tasks to further explore the underlying structure of these variables. The number of components to retain was determined using parallel analysis and visual inspection of the scree plot. We applied varimax rotation to obtain an orthogonal and interpretable solution. To assess robustness, we additionally performed an oblique rotation. After extracting the principal components, we examined their relationships with the navigational strategies questionnaires, including the NSQ14 and the three NSQ44 subscales. To account for multiple comparisons, we applied FDR correction.
