# Supplementary material for: Shared and distinct cognitive processes across multiple virtual navigation tasks
Source: iScience. 2026 Sep 24;29(10):117592. doi: 10.1016/j.isci.2026.117592 (PMC13634904; doi:10.1016/j.isci.2026.117592)
Supplement: Supplementary file 1 [file mmc1.pdf]

iScience, Volume *29*

Supplemental information

Shared and distinct cognitive processes across multiple virtual navigation tasks

Varnan Chandreswaran, David Stawarczyk, Anne Bierbrauer, David Clewett, Iva Brunec, Gillian Coughlan, Lila Davachi, Hugo Spiers, Michael Hornberger, Marta Silva, Lluis Fuentemilla, Morgan Barense, Jason Ozubko, Lukas Kunz, Dorothea Pink, Nikolai Axmacher, and Hui Zhang

Supplementary Material

Supplementary Figures

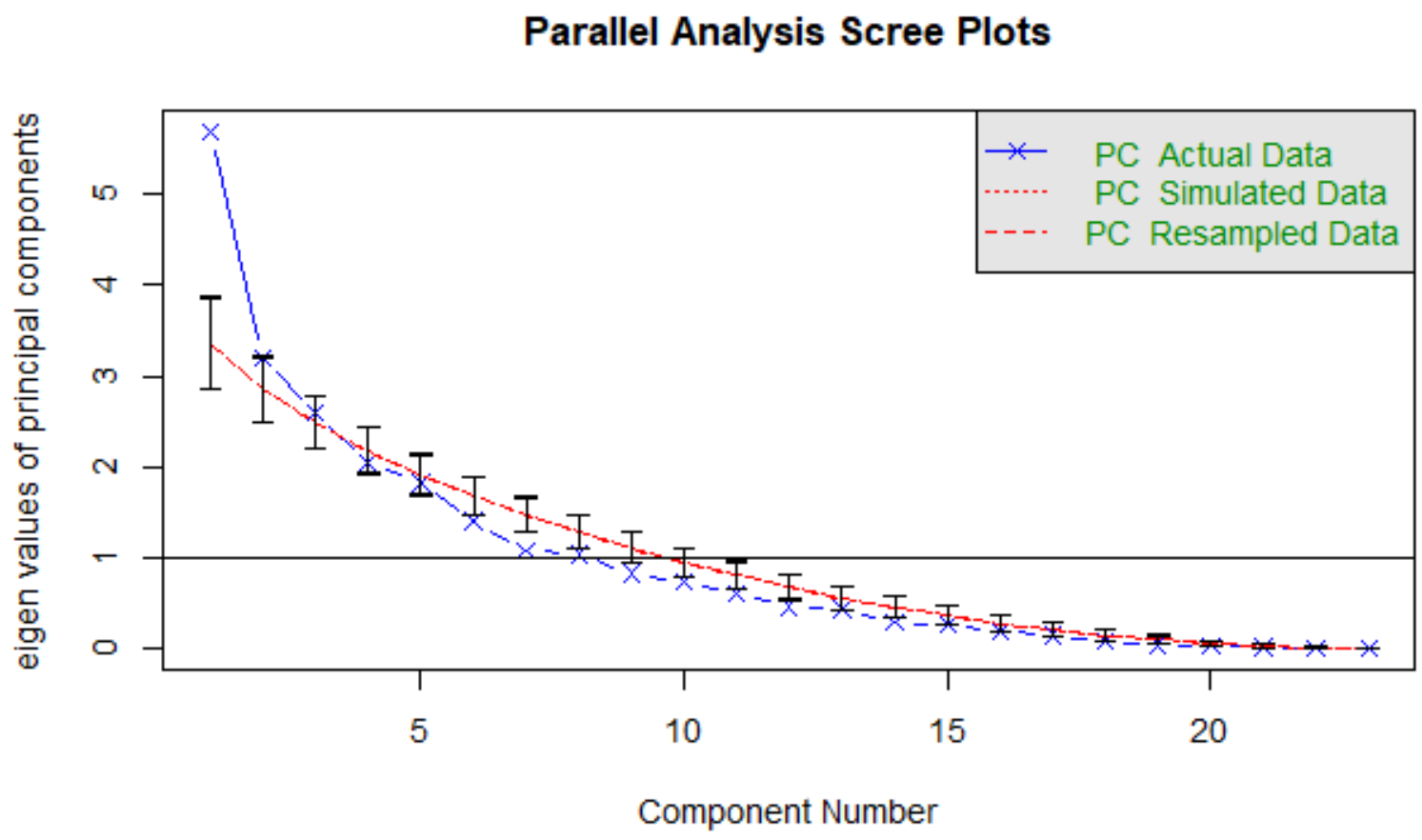

Figure S1. Parallel analysis scree plot for the principal component analysis of navigation variables. The plot shows eigenvalues of the observed data (blue line) together with eigenvalues obtained from randomly simulated datasets (green dashed line) and from resampled datasets based on the observed data (red dashed line). Error bars represent the variability of the simulated eigenvalues across 1,000 iterations.

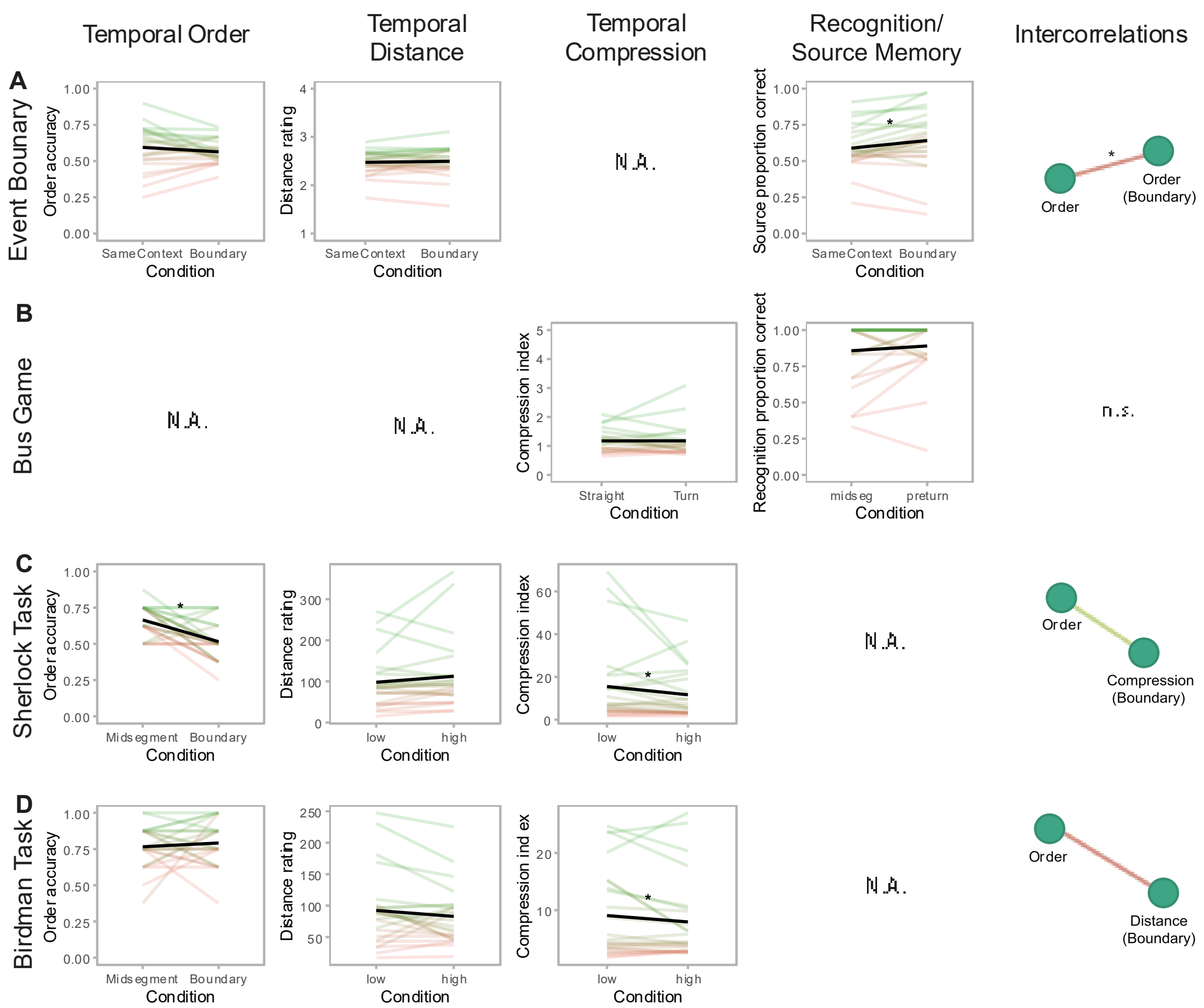

Figure S2. Predictors of performance across event segmentation tasks. Each slope in the individual plots represents a single subject's performance, with the color gradient reflecting overall task performance, ranging from green (best performance) to red (worst performance). Panel (A) shows results from the Auditory Event Boundary Task, (B) from the Bus Game, (C) from the Sherlock Task, and (D) from the Birdman Task. The plots display how performance is influenced by different conditions: temporal order (leftmost column), temporal distance (second column), temporal compression (third column), and recognition/source memory (fourth column). The rightmost column shows significant FDR-corrected intercorrelations between the performance metrics and boundary effects of each task. N.A. indicates that a measure was not available; n.s. indicates a non-significant result. Significant differences and correlations are marked with an asterisk ($p < .05$).

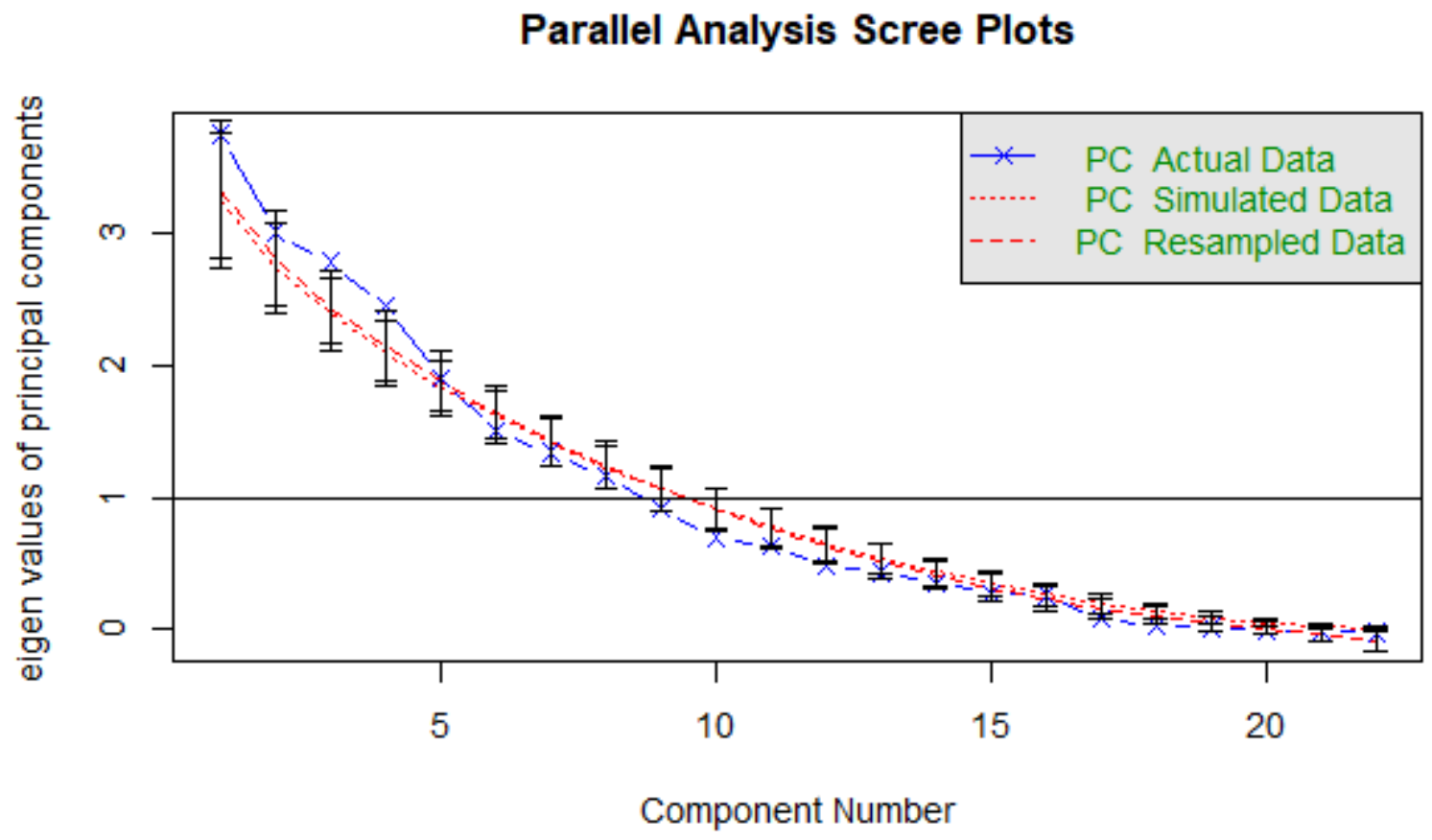

Figure S3. Parallel analysis scree plot for the principal component analysis of episodic memory variables. Eigenvalues of the observed data (blue line) are compared with eigenvalues derived from randomly simulated datasets (green dashed line) and resampled datasets based on the observed data (red dashed line). Error bars indicate variability across 1,000 iterations. Components with observed eigenvalues exceeding those expected from simulated data are considered meaningful according to the parallel analysis criterion.

Supplementary Tables

Table S1. Correlation matrix of Arena Game variables.

**Variable**	**1**	**2**	**3**	**4**	**5**
1. Performance	—				
2. Distance to Center	−.54*	—			
3. P(Trial)	.76*	-.52*	—		
4. P(Goal Distance to Center)	.61*	-.55*	.53*	—	

5. P(Goal Distance from Start)	.57*	-.52*	.56*	.68*	—

Note: The table reports Spearman correlation coefficients. Significant associations after FDR correction are indicated by an asterisk ($p < .05$).

Table S2. Correlation matrix of Island Game variables.

Variable	1	2	3	4	5
1. Performance Teleportation	—				
2. Performance Lure	.64*	—			
3. P(Goal Distance to Center)	.41*	.24	—		
4. P(Distance to Center)	.85*	.53*	.31	—	
5. P(Goal Distance from Start)	.69*	.33	.63*	.60*	—

Note: The table reports Spearman correlation coefficients. Significant associations after FDR correction are indicated by an asterisk ($p < .05$).

Table S3. Correlation matrix of Apple Game variables.

Variable	1	2	3	4	5	6
1. Subtask	—					
2. Performance PPI	.74*	—				
3. Performance LPI	.28	.81*	—			
4. Distance to Landmark	-.16	-.32	-.32	—		
5. P(Distance to Landmark)	-.09	.18	.39	-.19	—	
6. P(Goal Distance to Landmark)	-.03	-.10	-.16	.49	.18	—

Note: The table reports Spearman correlation coefficients. Significant associations after FDR correction are indicated by an asterisk (p < .05).

Table S4. Correlation matrix of Sea Hero Quest variables.

Variable	1	2	3	4	5
1. Performance	—				
2. Near Boundary	.13	—			
3. Performance Flare	.00	.01	—		
4. P(Near Boundary)	.06	.30	-.09	—	
5. P(Number of Checkpoints)	.11	.05	.51	-.33	—

Note: The table reports Spearman correlation coefficients. Significant associations after FDR correction are indicated by an asterisk (p < .05).

Table S5. Correlation matrix of performance variables.

Variable	1	2	3	4	5	6	7
1. ARG Performance	—						
2. AG Performance PPI	.31	—					
3. AG Performance LPI	.43	.81*	—				
4. IG Performance Teleportation	.38	.59*	.49	—			
5. IG Performance Lure	.40	.69*	.52*	.65*	—		
6. SHQ Performance	.05	.14	.21	-.07	.11	—	
7. SHQ Performance Flare	.14	.28	.25	.11	.16	.00	—

Note: The table reports Spearman correlation coefficients. Significant associations after FDR correction are indicated by an asterisk (p < .05). ARG = Arena Game; AG = Apple Game; IG = Island Game; SHQ = Sea Hero Quest; PPI = pure path integration; LPI = landmark-supported path integration.

Table S6. Rotated component loadings for navigation metrics (oblimin rotation).

Navigational Metrics	Component loading	
	1	2
Arena Game		
Performance	**.65**	.09
Distance to Center	**−.85**	−.01
P(Trial)	**.58**	.23
P(Distance to Center)	.12	**.41**
P(Goal Distance from Start)	**.75**	−.14
P(Goal Distance to Center)	**.70**	−.13
Apple Game		
Performance without Landmark (PPI)	−.16	**.92**
Performance with Landmark (LPI)	−.07	**.77**
Distance to Landmark	**.62**	**−.35**
P(Goal Distance to Landmark)	**.50**	−.22
P(Goal Distance from Start)	.13	.11
P(Distance to Landmark)	.07	.06
Island Game		
Performance (Continuous)	.15	**.80**
Performance (Teleport)	**.51**	**.62**
Distance to Center	**.54**	.09
P(Distance to Center)	**.46**	**.46**
P(Goal Distance to Center)	.41	.28
P(Goal Distance from Start)	**.57**	**.36**
Sea Hero Quest		
Performance Wayfinding	−.27	.17
Performance Flare	−.11	**.41**
Near Boundary	.02	**-.60**
P(Near Boundary)	.07	**−.32**
P(Path Distance)	-.23	.21

Note: Loadings are from a principal component analysis on the correlation matrix with oblimin rotation. Loadings with absolute values ≥ .30 are shown in bold.

Table S7. Correlation matrix of principal components with self-reported navigation preferences.

Variable	1	2	3	4	5	6
1. PC1	—					
2. PC2	−.38	—				
3. NSQ14	-.06*	.39	—			
4. NSQ44 - Allocentric	.28	.18	.44*	—		
5. NSQ44 - Egocentric	−.22	.31	.43*	.64*	—	

6. NSQ44 – Route/Procedural	.39	−.56*	-.40	-.43	.56*	—

Note: The table reports correlation coefficients among the two principal component scores (PC1 and PC2), the NSQ-14 composite score, and the three NSQ-44 subscales (Allocentric, Egocentric and Route/Procedural). Correlation type was selected based on variable distributions, with Pearson applied to normally distributed pairs and Spearman otherwise. Significant associations after FDR correction are indicated by an asterisk (p < .05).

Table S8. Correlation matrix of Auditory Event Boundary variables.

Variable	1	2	3	4	5	6
1. Order	—					
2. Distance	.08	—				
3. Order Boundary Effect	-.71*	-.12	—			
4. Distance Boundary Effect	-.04	.26	.10	—		
5. Source	.29	.38	-.38	.15	—	
6. Source Boundary Effect	-.11	.10	-.10	.22	.25	—

Note. *The table reports Spearman correlation coefficients. Significant associations after FDR correction are indicated by an asterisk (p < .05).*

Table S9. Correlation matrix of Bus Game variables.

**Variable**	**1**	**2**	**3**	**4**
1. Compression	—			
2. Compression Boundary Effect	-.02	—		
3. Recognition	.36	-.05	—	
4. Recognition Boundary Effect	.11	.04	-.28	—

Note. *The table reports Spearman correlation coefficients. No associations survived FDR correction at p < .05.*

Table S10. Correlation matrix of Birdman Movie Task variables.

**Variable**	**1**	**2**	**3**	**4**	**5**
1. Compression	—				
2. Distance	.09	—			
3. Compression Boundary Effect	-.33	-.29	—		
4. Distance Boundary Effect	-.12	.02	.09	—	
5. Order	-.11	-.16	.43	-.01	—
6. Order Boundary Effect	-.13	.11	-.14	-.31	.23

Note. *The table reports Spearman correlation coefficients. No associations survived FDR correction at p < .05.*

Table S11. Correlation matrix of Sherlock Movie Task variables.

Variable	1	2	3	4	5	6
1. Compression	—					
2. Distance	-.15	—				
3. Compression Boundary Effect	-.37	-.09	—			
4. Distance Boundary Effect	.20	-.37	-.49	—		
5. Order	-.12	-.20	.56	-.31	—	
6. Order Boundary Effect	.15	-.30	.03	.18	.07	—

Note. *The table reports Spearman correlation coefficients. No associations survived FDR correction at $p < .05$.*

Table S12. Descriptive statistics for navigation task performance.

Task	N	M	SD	Median	IQR	Min	Max
Apple Game	24	2767.94	605.15	2698.56	699.64	1621.57	3953.41
Arena Game	24	2270.73	678.97	2269.95	795.78	1195.39	3780.17
Island Game	24	6.75	1.55	6.94	1.80	3.38	9.67
Sea Hero Quest	24	2.00	0.20	2.04	0.29	1.56	2.31

Table S13. Correlation matrix of all navigation variables.

Variable	1	2	3	4	5	6	7	8	9	10	11	12	13	14	15	16	17	18	19	20	21	22
1. SHQ Performance	—																					
2. SHQ Near Boundary	.13	—																				
3. SHQ Performance Flare	.00	.01	—																			
4. SHQ Cor Near Boundary	.06	.30	−.09	—																		
5. SHQ Cor Checkpoints	.11	.05	.51	−.33	—																	
6. AG Performance (PPI)	.14	−.38	.28	−.19	.03	—																
7. AG Performance (LPI)	.21	−.15	.25	−.14	.08	.81	—															
8. AG Dist to Landmark	−.27	−.16	−.09	−.15	−.10	−.32	−.32	—														
9. AG Cor Dist to Landmark	−.13	−.15	.12	−.17	.11	.18	.39	−.19	—													
10. AG Cor Goal to Landmark	−.27	−.23	−.24	−.11	.01	−.10	−.16	.49	.18	—												
11. AG Cor Incoming	.30	−.01	−.28	−.19	.11	.17	.04	−.21	.01	.14	—											
12. ARG Performance	.05	−.07	.14	.12	−.21	.31	.43	.16	.18	.24	−.16	—										
13. ARG Dist to Center	.06	.00	.00	−.16	.35	−.07	−.15	−.35	.03	−.12	−.17	−.55	—									
14. ARG Cor Trial	.01	−.07	.43	.09	.06	.30	.25	.12	.19	.11	.07	.76	−.52	—								
15. ARG Cor Dist to Center	.17	−.15	.26	−.28	.41	.14	−.01	.27	.09	.23	.29	−.11	.03	.24	—							
16. ARG Cor Target to Center	.08	.03	.09	−.10	−.07	.11	.20	.13	.19	.25	.33	.61	−.55	.53	.00	—						
17. ARG Cor Incoming	.40	.06	.13	.10	−.00	−.05	−.03	.26	−.13	.28	.28	.57	−.52	.56	.23	.68	—					
18. IG Dist to Center	.13	.07	−.11	−.16	.17	−.23	−.12	.37	−.20	.09	.02	−.18	−.05	−.18	.12	.21	.18	—				
19. IG Performance (Teleport)	−.07	−.29	.11	−.18	−.20	.59	.49	.10	−.09	−.05	.20	.38	−.51	.40	.16	.22	.19	−.26	—			
20. IG Performance (Lure)	.11	−.46	.16	−.36	−.06	.69	.52	−.16	.32	.07	.16	.40	−.22	.45	.35	.24	.13	−.17	.65	—		
21. IG Cor Target to Center	−.13	−.47	−.11	−.16	.08	.16	.07	.62	−.10	.31	.01	.07	−.37	.12	.24	−.05	.03	.26	.41	.25	—	
22. IG Cor Dist to Center	−.15	−.14	−.03	−.18	−.37	.37	.36	.22	.02	.02	.09	.31	−.50	.22	.25	.15	.15	−.26	.85	.53	.31	—
23. IG Cor Incoming	−.15	−.38	.01	−.09	−.06	.38	.40	.39	−.06	.24	−.02	.33	−.48	.21	.04	.15	.26	.15	.69	.33	.63	.60

Note. *The table reports Spearman correlation coefficients. Significant associations after FDR correction are indicated by an asterisk (p < .05). ARG = Arena Game; AG = Apple Game; IG = Island Game; SHQ = Sea Hero Quest; PPI = pure path integration; LPI = landmark-supported path integration.*
